# The Transcriptional Landscape of Microglial Genes in Aging and Neurodegenerative Disease

**DOI:** 10.3389/fimmu.2019.01170

**Published:** 2019-06-04

**Authors:** Luke W. Bonham, Daniel W. Sirkis, Jennifer S. Yokoyama

**Affiliations:** Department of Neurology, Memory and Aging Center, University of California, San Francisco, San Francisco, CA, United States

**Keywords:** microglia, Alzheimer's disease, genetics, TMEM119, cell-type profiling, frontotemporal dementia, autoimmune disease, RNAseq

## Abstract

Microglia, the brain-resident myeloid cells, are strongly implicated in Alzheimer's disease (AD) pathogenesis by human genetics. However, the mechanisms by which microglial gene expression is regulated in a region-specific manner over the course of normal aging and in neurodegenerative disease are only beginning to be deciphered. Herein, we used a specific marker of microglia (*TMEM119*) and a cell-type expression profiling tool (*CellMapper*) to identify a human microglial gene expression module. Surprisingly, we found that microglial module genes are robustly expressed in several healthy human brain regions known to be vulnerable in AD, in addition to other regions affected only later in disease or spared in AD. Surveying the microglial gene set for differential expression over the lifespan in mouse models of AD and a related tauopathy revealed that the majority of microglial module genes were significantly upregulated in cortex and hippocampus as a function of age and transgene status. Extending these results, we also observed significant upregulation of microglial module genes in several AD-affected brain regions in addition to other regions using postmortem brain tissue from human AD samples. In pathologically confirmed AD cases, we found preliminary evidence that microglial genes may be dysregulated in a sex-specific manner. Finally, we identified specific and significant overlap between the described microglial gene set—identified by unbiased co-expression analysis—and genes known to impart risk for AD. Our findings suggest that microglial genes show enriched expression in AD-vulnerable brain regions, are upregulated during aging and neurodegeneration in mice, and are upregulated in pathologically affected brain regions in AD. Taken together, our data-driven findings from multiple publicly accessible datasets reemphasize the importance of microglial gene expression alterations in AD and, more importantly, suggest that regional and sex-specific variation in microglial gene expression may be implicated in risk for and progression of neurodegenerative disease.

## Introduction

Alzheimer's disease (AD) is a devastating neurodegenerative disorder involving the progressive loss of memory and cognitive abilities. Discoveries over the last decade suggest that many, perhaps even a majority, of the genes contributing risk to AD are expressed primarily by microglia (1, 2), the resident myeloid cells of the brain parenchyma. Furthermore, work from the past few years demonstrates that microglial gene expression changes during aging and may contribute to risk for AD (3) as well as autoimmune disorders of the central nervous system (CNS) such as multiple sclerosis [MS; reviewed in (4)] and lupus (5), but precisely how these cells contribute to risk for autoimmune disorders or AD remains unclear. Although AD is not considered a classical autoimmune disease, genetic pleiotropy studies have found striking genetic overlap between AD and several autoimmune disorders, including psoriasis and Crohn disease (6). Beyond these findings, a specific class II human leukocyte antigen (HLA)-DR haplotype, DR15, has been suggested to impart risk for both AD and MS (7, 8). These findings suggest that microglia may represent a common cellular link between canonical autoimmune disorders and AD, but it remains unclear whether shared or distinct microglial activities underlie the etiologies of these disorders.

To better understand the role of microglia in normal aging as well as in AD, robust and selective markers enabling unambiguous identification of microglia are required. Prior to the development of single-cell sequencing technologies, it was difficult to systematically identify and characterize *bona fide* microglia—as opposed to other myeloid cells, including perivascular macrophages and infiltrating monocyte-derived macrophages—at the molecular level and across multiple brain regions throughout the lifespan. However, advances within the last 5 years have enabled the identification of highly specific microglial markers not shared by other myeloid cell populations. In particular, the identification of the transmembrane protein TMEM119 as a specific marker of microglia has enabled global gene expression profiling of highly pure preparations of microglia (9). Beyond the identification of specific microglial markers, advances in cell-type expression profiling have enabled the identification of cell-type-specific gene expression profiles (10). These techniques do not require microdissection of the target cell type, but rather rely on a single cell-restricted marker (e.g., *TMEM119*) to reveal additional genes expressed by a given cell type.

In this study, we used multiple publicly available datasets to explore microglial gene expression in both healthy aging and disease. We leveraged the specificity of *TMEM119* expression and the *CellMapper* tool (10) to identify robustly co-expressed genes in human microglia. We utilized this microglial gene expression profile to explore how microglial genes are expressed in both healthy human aging and neurodegenerative disease. Our data-driven, expression-based microglial gene set demonstrated significant upregulation during normal aging and dysregulation in regions known to show atrophy in AD along with other brain regions. Further, it overlapped with genes implicated in risk for AD. Taken together, our results demonstrate that co-expressed microglial genes display regional heterogeneity in terms of expression level, are found at high levels in brain regions vulnerable in AD, and are significantly dysregulated in neurodegenerative disease. These results stengthen the known association of microglia with neurodegenerative disease and suggest that brain regions selectively vulnerable in AD may show greater numbers of microglia even in the healthy brain.

## Methods

### Identification of Microglia-Specific Genes

We identified microglia-specific genes using previously described techniques to identify and characterize cell-specific expression profiles for rare or difficult-to-isolate cell types (10). Briefly, these methods take advantage of inherent cell type variability within a given bulk tissue sample set (e.g., undissociated brain tissue) by identifying genes with similar expression profiles. Although many techniques are available to identify a cell-specific expression profile, they often require multiple cell type markers and large training datasets. The technique we chose, *CellMapper*, requires only a single cell marker and a smaller training dataset than other techniques, and enables analysis of expression data derived from bulk brain tissue samples (10). These characteristics are especially important for native microglia in brain tissue—publicly available brain tissue samples are limited and many native microglial markers are shared with other ostensibly similar cell populations (e.g., infiltrating peripheral macrophages).

Validated cell markers specific for brain-resident microglia remained, until the last 5 years, elusive and difficult to confirm. There are multiple proposed markers, including *P2ry12, Fcrls, Siglec-H, Olfml3*, and *Tmem119* (9, 11, 12), but several of these markers are not yet validated or derived from models of neurodegeneration. We analyzed *TMEM119* in bulk human brain tissue data from the Allen Brain Institute (13) because it is a highly specific and well-validated marker of brain-derived microglia in both mice and humans.

### Gene Set Expression Enrichment Analyses in Normal Human Tissue

We next determined the spatial patterns in microglial gene expression using tissue samples from the Allen Brain Institute. These analyses relied on one dataset available to the public (13) through the Allen Brain Institute (human.brain-map.org). The dataset included 6 adult control brain samples (H0351.2001, H0351.2002, H0351.1009, H0351.1012, H0351.1015, and H0351.106) finely dissected as described in the documentation available at http://help.brain-map.org/display/humanbrain/Documentation.

We tested whether microglial genes were regionally enriched by using novel methods to compare the number of query genes expressed above baseline in each region (14). Briefly, enrichment was calculated using the number of query genes expressed above baseline for each tissue type compared to the background gene expression for the aforementioned region. Statistical significance was calculated using a bootstrapping procedure comparing the provided gene list against the overlap occurring in randomly generated gene sets. Multiple testing correction was conducted using the FDR technique (15).

### Differential Expression Analyses in Mouse Models of Neurodegenerative Disease

To better understand the dynamics of the microglial gene module in both healthy aging and disease, we examined its expression in two mouse models of neurodegenerative disease alongside wild type mice. We utilized data from the Mouseac project, which includes brain tissue samples from mouse models of neurodegenerative disease and wild type (WT) mice of the same background strain at varying ages (i.e., 8, 16, 32, and 72 weeks). The Mouseac project has been described in detail elsewhere (16). Briefly, samples were collected from three brain regions (cortex, hippocampus, and cerebellum) from wild-type, TASTPM (TAS10 × TPM AD mouse models; APPswe × PS1.M1466V), and P301L-tau transgenic mice, which model a related neurodegenerative disorder, frontotemporal lobar degeneration (FTLD). Amongst the mouse models of AD available from the Mouseac project, we chose to analyze the TASTPM model because it involves two mutant, disease-causing transgenes (*APP* and *PS1*) identified in human familial AD cases and demonstrates a more severe pathological burden than either mutation alone. In addition, we analyzed the P301L mouse model of FTLD. Both mouse models are known to demonstrate a proinflammatory phenotype (17, 18). Of note, both heterozygous and homozygous carriers of the TASTPM transgenes were available for analysis, while only heterozygous carriers of the P301L-tau transgene were available. For the TASTPM mouse data, we analyzed heterozygous and homozygous mice together, accounting for gene dosage.

Gene expression was measured using microarrays (Illumina Ref8 v2) and processed by the Mouseac project staff. Briefly, raw expression levels were normalized using a log_2_ transformation and quantile normalization was performed for all samples together. An individual probe was excluded if the *p*-value for detection was >0.05 in >50% in a given group's samples at any age. Additionally, samples were excluded if <95% of the probes for a given gene were detected.

We tested whether microglial gene expression in AD mouse models varied from control tissue with respect to both age and brain region using ANOVA.

### Differential Expression in Pathologically Diagnosed Human AD Tissue

RNA sequencing (RNAseq) data from pathologically confirmed AD cases was used to explore whether areas that display enriched microglial gene expression in the healthy human brain also show enhanced expression in AD cases relative to pathologically normal controls. This data was obtained through the Accelerating Medicines Partnership—Alzheimer's Disease (AMP-AD) portal. Samples used for this portion of the study included those from the Mount Sinai School of Medicine (MSSM) Brain Bank (19), Mayo Clinic Brain Bank (20), and Religious Orders Study and Memory and Aging Project (ROSMAP) Study (21, 22). In aggregate, over 500 individuals were included in these analyses; cohort characteristics and sample distribution by region are available in Table 1. Data from the three brain banks' collective samples was reprocessed and harmonized using a consensus toolset at the Mount Sinai Icahn School of Medicine Minerva HPC system. The results are accessible online through Synapse (ID # syn14237651). Technical details describing the reprocessing and analysis are explained in detail online through Synapse (https://www.synapse.org/#!Synapse:syn14237651). The resulting dataset includes tissue samples from many relevant brain regions, including some impacted early in AD (e.g., superior temporal cortex, parahippocampal gyrus, inferior frontal gyrus) as well as regions not impacted until much later in AD (e.g., cerebellum and frontal pole). In all analyses, the effect of diagnosis on gene expression was tested using linear regression controlling for biological factors such as sex, age, postmortem interval, and technical confounders accounting for more than 1% of variance of the principal components. To illustrate the results of our analyses in multiple brain regions in the context of atrophy patterns typically seen in AD, we created an atrophy map using voxel-based morphometry. The map included data from 120 individuals (60 clinically diagnosed AD cases compared to 60 normal controls). All individuals were seen at the UCSF Memory and Aging Center and scanned on a 3 Tesla scanner as previously described (23). The images were processed using SPM12 (24, 25) and analyzed as previously described (26).

**Table 1:** AD cohorts used for differential expression analysis.

			**Sample count by diagnosis**			
**Study**	**Total cohort size**	**Tissue type**	**AD**		**Control**	
			**Female**	**Male**	**Female**	**Male**
Mayo	179	CBE	47	32	35	37
		TCX	49	31	35	36
MSSM	164	FP	63	27	23	22
		IFG	55	24	17	20
		PHG	47	18	18	20
		STG	57	28	20	17
ROSMAP	241	DLPFC	109	46	47	39

*For each cohort, the total number of participants is provided along with sample counts for each tissue type grouped by diagnosis and sex. Note, not all participants provided samples for all brain regions. RNA expression data from the cohorts shown above corresponds to differential expression analyses presented in Figure 4, Table S1, Figure S2. For additional information, please see www.synapse.org under entry syn14237651. CBE, Cerebellum; DLPFC, Dorsolateral Prefrontal Cortex; FP, Frontal Pole; IFG, Inferior Frontal Gyrus; MAYO, Mayo Clinic Brain Bank Data (see syn14237651 for additional details); MSSM, Mount Sinai Brain Bank study (see syn14237651 for additional details); PHG, Parahippocampal Gyrus; ROSMAP, Religious Orders Study and Memory and Aging Project study (see syn14237651 for additional details); STG, Superior Temporal Gyrus; TCX, Temporal Cortex*.

Given that AD shows a sex-specific incidence and findings indicating that microglia show sex-specific differentiation and gene expression profiles in adult mice (27–29), we also examined whether there was a statistical interaction between AD diagnosis and sex (i.e., Gene expression = Diagnosis × Sex + covariates), using the covariate selections described above.

### Microglial Gene Enrichment in Alzheimer's Disease Genome-Wide Association Study (GWAS) Data

We tested whether our microglial gene set showed specific enrichment for AD risk genes using FUMA GWAS (Functional Mapping and Annotation of Genome-Wide Association Studies), a platform developed to characterize and interpret the results of genetic analyses (30). Briefly, FUMA compares a user's list of submitted genes to publicly reported disease-associated genes (e.g., AD risk genes) and computes an associated *p*-value using hypergeometric testing. The background GWAS datasets used for this analysis come from the NHGRI-EBI catalog of published genome-wide association studies (https://www.ebi.ac.uk/gwas/), which contains over 2,500 publications and over 24,000 single nucleotide polymorphism (SNP) trait associations (31). We tested the 30 microglial genes identified using *CellMapper* against all available genes and associations in the catalog (a total of over 3,000 unique diseases and traits).

### Statistical Analyses

All statistical analyses were performed using R (version 3.3.3) unless otherwise specified.

### Ethics Statement

This study was carried out in accordance with the recommendations of University of California, San Francisco Committee on Human Research. The protocol was approved by the University of California, San Francisco Committee on Human Research. All subjects gave written informed consent in accordance with the Declaration of Helsinki.

## Results

### TMEM119 Identifies 30 Additional Microglial Genes

Using *CellMapper* and *TMEM119* as a marker of native microglia, we identified 30 additional genes associated with a microglial gene expression profile (Table 2; p_FDR_ < 0.05). Some of these genes, like *TREM2*, are known to be expressed by microglia and are associated with neurodegenerative diseases (32–34). Others, like *SUCNR1*, have established functions in inflammatory pathways, but are not known to be associated with neurodegenerative diseases (35). In Table 3, we provide a summary describing the protein product encoded by each gene, its cellular localization, regions of expression in the brain, and potential roles in normal aging as well as neurodegenerative disease. Using a stricter significance threshold of p_FDR_ < 0.01, 11 genes remained significant (*IGSF6, ADORA3, ALOX5AP, CSF2RA, HPGDS, P2RY13, ACY3, SUSD3, SASH3, TBXAS1*, and *RASAL3*). In the analyses that follow, we evaluated the entire 30 gene set when possible, omitting specific microglial genes only when expression data was not available.

**Table 2:** Microglial genes identified by *TMEM119* expression profile.

**Gene symbol**	**Name**
*ACY3*	Aminoacylase 3
*ADAM28*	ADAM metallopeptidase domain 28
*ADORA3*	Adenosine A3 receptor
*ALOX5AP*	Arachidonate 5-lipoxygenase activating protein
*C1QB*	Complement C1q B chain
*C3*	Complement C3
*CD33*	CD33 molecule
*CD84*	CD84 molecule
*CIITA*	Class II major histocompatibility complex transactivator
*CPED1*	Cadherin like and PC-esterase domain containing 1
*CSF2RA*	Colony stimulating factor 2 receptor alpha subunit
*DHRS9*	Dehydrogenase/reductase 9
*FCER1G*	Fc fragment of IgE receptor Ig
*FYB*	FYN binding protein
*GPR34*	G protein-coupled receptor 34
*HPGDS*	Hematopoietic prostaglandin D synthase
*IGSF6*	Immunoglobulin superfamily member 6
*LAPTM5*	Lysosomal protein transmembrane 5
*LY86*	Lymphocyte antigen 86
*P2RY13*	Purinergic receptor P2Y13
*RASAL3*	RAS protein activator like 3
*SASH3*	SAM and SH3 domain containing 3
*SELPLG*	Selectin P ligand
*SPN*	Sialophorin
*SUCNR1*	Succinate receptor 1
*SUSD3*	Sushi domain containing 3
*SYK*	Spleen associated tyrosine kinase
*TBXAS1*	Thromboxane A synthase 1
*TLR7*	Toll like receptor 7
*TREM2*	Triggering receptor expressed on myeloid cells 2

*Using TMEM119 as a marker of native microglia, we identified a set of 30 additional genes whose expression profile suggested relevance to microglial function. The names of these genes along with their associated gene symbol are provided*.

**Table 3:** Protein function, cellular location, and disease associations for microglial genes.

**Gene symbol**	**Protein function**	**Cellular locations of protein**	**Brain location of protein**	**Notes**	**References**
*ADAM28*	Modulate cell-cell and cell-matrix interactions; implicated in neurogenesis	Mitochondria; plasma membrane	Cerebral cortex	Expression lower in AD CSF	(36–39)
*CD33*	Cell-cell interactions; maintenance of resting state in immune cells	Nucleus; plasma membrane	Cerebral cortex	Known AD risk gene	(37–40)
*CD84*	Cell-cell interactions; modulate activation and differentiation of innate and adaptive immune system	Plasma membrane	Not yet determined	Upregulated during plaque development in mouse models of AD	(37, 41, 42)
*FYB*	Adapter protein of FYN and LCP2 signaling cascades; modulate expression of IL2	Cytosol	Cerebral cortex		(37–39)
*FCER1G*	Tyrosine kinase-based activation motif for transduction of immune activation signals	Plasma membrane	Cerebral cortex; hippocampus; caudate; cerebellum	RNA and protein expression noted to be discrepant. Upregulated in AD cases	(37–39, 43, 44)
*GPR34*	Orphan Gi protein-coupled receptor implicated in immune response, receptor for short chain fatty acids	Nucleus; cytosol	Cerebral cortex; hippocampus; caudate; cerebellum	Appears to have multi-pass membrane component	(37–39, 45, 46)
*RASAL3*	Negative regulation of RAS signaling	Cytoplasm near the plasma membrane	Cerebral cortex		(37, 39, 47)
*SASH3*	Signaling adapter protein in lymphocytes	Plasma membrane	Cerebral cortex		(37–39)
*ADORA3*	Adenosine receptor	Plasma membrane	None	Primarily expressed in lung, liver, kidney, and heart. Downregulated in aging	(37, 48, 49)
*ACY3*	Deacetylation of mercapturic acids, classically associated with kidney proximal tubule and gastrointestinal tract function	Plasma membrane; cytosol	Unspecified, but present at low levels in mouse	See Pushkin et al. (50) for brain expression data	(37, 50, 51)
*ALOX5AP*	Leukotriene synthesis and promotion of inflammatory responses	Nuclear envelope; endoplasmic reticulum	Cerebral cortex	Associated with stroke and AD	(37, 39, 52–54)
*CPED1*	Not well understood. Multiple likely protein products.	Nucleus; endoplasmic reticulum	Hippocampus; caudate		(37–39, 55)
*CIITA*	Required for transcriptional activity of Class II MHC receptor and Class I MHC receptor to a lesser extent	Nucleus	Cerebral cortex; hippocampus; caudate; cerebellum		(37–39)
*CSF2RA*	Controls production, differentiation, and function of granulocytes and macrophages	Extracellular; plasma membrane	Detected throughout the CNS	Reduced protein expression in hippocampus of human AD cases	(37, 56–58)
*C1QB*	Initiation of the complement cascade	Extracellular; blood microparticle	Cerebral cortex; cerebellum		(37, 39, 59, 60)
*C3*	Activation of the classical and alternative complement pathways	Plasma membrane; extracellular; endoplasmic reticulum; lysosome	Hippocampus	Primarily extracellular; broadly implicated in AD	(37, 59, 61–63)
*DHRS9*	Steroid and retinoid synthesis	Endoplasmic reticulum	Caudate; cerebellum		(37, 39, 64)
*HPGDS*	Prostaglandin synthesis	Cytosol	Frontal cortex; hippocampus	Expression localizes to microglia and astrocytes in human AD cases	(37, 65, 66)
*IGSF6*	Not well understood. Associated with transmembrane signaling receptor activity.	Plasma membrane	Not yet determined	Associated with inflammatory bowel disease	(37, 67, 68)
*LY86*	Innate immune response to lipopolysaccharide and cytokine production	Plasma membrane	Cerebral cortex		(37, 39, 69)
*LAPTM5*	Thought to play a role in embryogenesis and hematopoietic cell function	Cytosol	Cerebral cortex; hippocampus; caudate; cerebellum		(37–39)
*P2RY13*	ADP receptor for Gi coupled signaling pathways	Plasma membrane	Cerebral cortex; hippocampus	Downregulated in aging	(37, 39, 48)
*SELPLG*	Glycoprotein receptor for P, E, and L selectins	Plasma membrane	Not detected		(37, 70, 71)
*SPN*	Sialophorin involved in T-cell functions such as activation, proliferation, differentiation, trafficking, and migration	Golgi apparatus, plasma membrane, cell junctions	Hippocampus; temporal cortex	Downregulated in AD	(37, 38, 72, 73)
*SYK*	Non-receptor tyrosine kinase mediating signal transduction that modulates adaptive and innate immunity	Plasma membrane; nucleus	Cerebral cortex; hippocampus; caudate; cerebellum	Syk expression is activated by amyloid and tau accumulations	(37, 74, 75)
*SUCNR1*	Receptor for succinate, involved in the promotion of hematopoietic progenitor cell development	Plasma membrane	Not yet determined	Associated with an anti-inflammatory phenotype	(37, 39, 76)
*SUSD3*	Not well understood. Implicated in estrogen-dependent cell proliferation in breast cancer	Nucleus	Cerebral cortex; hippocampus; caudate; cerebellum		(37–39)
*TBXAS1*	Cytochrome p450 member, catalyzes the conversion of prostaglandin H2 to thromboxane A2	Intracellular vesicles	Cerebral cortex; hippocampus; caudate; cerebellum	Demonstrates coordinated expression changes during development and aging	(37–39, 77)
*TLR7*	Toll-like receptor implicated in pathogen recognition and innate immunity	Cytoplasm; plasma membrane; endolysosome	Hippocampus; neocortex	Enhances microglial amyloid uptake during early AD	(37, 78–82)
*TREM2*	Membrane protein forming a signaling complex with TYRO, functions in immune response and triggers inflammatory chemokines	Plasma membrane; lysosomes	Cerebral cortex; hippocampus; caudate; cerebellum	Implicated as a risk factor in AD	(37, 39, 83)

*For each gene identified in CellMapper analyses, we used Uniprot (https://www.uniprot.org) to summarize the function of each microglial gene's corresponding protein product. We then utilized the Human Protein Atlas to identify the subcellular and brain locations of each protein product. Finally, we performed a targeted literature search for each protein to identify additional subcellular location or brain expression information as well as to find aging and neurodegenerative disease-relevant publications. CNS, Central nervous system; CSF, Cerebrospinal fluid; AD, Alzheimer's disease*.

### Microglial Genes Are Enriched in Healthy Human Temporal and Parietal Cortex, Basal Ganglia, and Brainstem Nuclei

**Figure 1:**
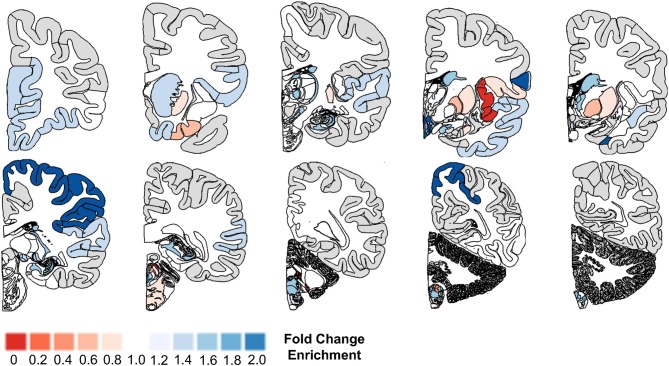
Microglial gene enrichment analysis in normal human brain. We utilized gene set enrichment analyses to test whether the 30 microglial genes we identified using cell-type profiling showed a tropism for specific brain regions. Our analyses suggest that there is diffuse involvement of multiple brain regions, but also that there is focal involvement of specific regions such as the frontal pole, temporal cortex, basal ganglia, parietal cortex, and brain stem nuclei. For additional details, please the manuscript text and methods described in Linker et al. (14).

Utilizing the microglial gene set identified above, we tested which brain regions demonstrate enriched microglial gene expression in finely-dissected healthy human tissue samples. Our analyses revealed significant enrichment in 48 out of 194 testable brain regions at a p_FDR_ < 0.05 (Table 4, Figure 1). Of note, enrichment was particularly notable in the parietal cortex, areas of the temporal cortex (e.g., temporal pole, parahippocampal gyrus, amygdalohippocampal transition), basal ganglia (e.g., putamen and globus pallidus), and brainstem nuclei (e.g., vestibular nuclei, pontine nuclei, and paraventricular nuclei) (Table 4, Figure 1). Although the frontal lobes showed comparably less enrichment relative to other brain regions, there was significant enrichment in the inferior frontal gyrus and frontal pole. Notably, both the cerebellum and occipital cortex showed minimal microglial gene enrichment.

**Table 4:** Regional gene set enrichment analyses in healthy human brain tissue.

**Structure**	**Fold change**	**Raw *p*-value**	**Adjusted *p*-value**
Corpus callosum	1.667	2.52E-13	4.86E-11
Temporal pole, right, medial aspect	1.404	3.10E-09	5.94E-07
Globus pallidus, internal segment, right	1.376	4.85E-09	9.27E-07
Principal sensory nucleus of trigeminal nerve, right	1.333	6.66E-08	1.27E-05
Parahippocampal gyrus, left, lateral bank of gyrus	1.136	1.75E-07	3.32E-05
Parolfactory gyri, left	1.259	1.94E-07	3.65E-05
Globus pallidus, external segment, right	1.280	2.04E-07	3.81E-05
Posterior orbital gyrus, right	1.200	2.05E-07	3.82E-05
Frontal pole, left, medial aspect	1.250	2.32E-07	4.29E-05
Subcallosal cingulate gyrus, left	1.173	2.58E-07	4.74E-05
Lateral group of nuclei, right, dorsal division	1.070	2.71E-07	4.97E-05
Paraterminal gyrus, right	1.161	3.39E-07	6.18E-05
Cochlear nuclei, left	1.232	6.52E-07	1.18E-04
Putamen, right	1.037	1.28E-06	2.31E-04
Short insular gyri, left	1.102	1.42E-06	2.54E-04
Temporal pole, left, inferior aspect	1.180	1.44E-06	2.56E-04
Parahippocampal gyrus, left, bank of the cos	1.223	1.49E-06	2.65E-04
Gyrus rectus, right	0.950	1.57E-06	2.77E-04
Lateral parabrachial nucleus, left	1.118	2.21E-06	3.87E-04
Superior frontal gyrus, right, medial bank of gyrus	0.903	6.95E-06	0.001
Lateral orbital gyrus, left	0.913	8.32E-06	0.001
Precentral gyrus, left, bank of the precentral sulcus	0.941	9.36E-06	0.002
Locus ceruleus, right	1.055	9.79E-06	0.002
Vestibular nuclei, left	1.013	1.31E-05	0.002
Pontine raphe nucleus	1.000	2.98E-05	0.005
Paraventricular nuclei, right of thalamus, right	0.987	3.92E-05	0.007
Middle frontal gyrus, left, inferior bank of gyrus	1.004	4.19E-05	0.007
Temporal pole, right, superior aspect	0.904	4.59E-05	0.008
Frontal pole, right, superior aspect	0.984	4.63E-05	0.008
Medial orbital gyrus, left	0.945	4.68E-05	0.008
Pontine nuclei, right	0.989	4.73E-05	0.008
Inferior rostral gyrus, right	0.948	4.91E-05	0.008
Frontal pole, left, inferior aspect	0.987	5.14E-05	0.008
Planum polare, right	0.968	5.22E-05	0.008
Frontal operculum, left	1.036	5.50E-05	0.009
Gigantocellular group, left	1.027	6.73E-05	0.011
Medial parabrachial nucleus,right	0.905	7.22E-05	0.011
Amygdalohippocampal transition zone, right	0.962	1.94E-04	0.030
Inferior olivary complex, left	0.860	1.97E-04	0.030
Superior rostral gyrus, left	0.925	2.20E-04	0.034
Lateral group of nuclei, left, ventral division	0.871	2.21E-04	0.034
Inferior frontal gyrus, opercular part, right	0.943	2.21E-04	0.034
Middle frontal gyrus, left, superior bank of gyrus	0.936	2.30E-04	0.035
Inferior frontal gyrus, orbital part, right	0.913	2.54E-04	0.038
Fusiform gyrus, left, bank of cos	0.925	2.55E-04	0.038
Cingulate gyrus, frontal part, left, inferior bank of gyrus	0.956	2.61E-04	0.039
Medial geniculate complex, right	0.860	2.68E-04	0.039
Midbrain raphe nuclei	0.933	2.90E-04	0.042

*Using BrainimageR (14), we conducted an unbiased analysis to explore the human brain regions where microglial genes were more highly expressed compared to background gene expression. Interestingly, microglial gene expression was enriched in multiple brain regions, some of which are implicated in Alzheimer's disease (AD), such as the parahippocampal gyrus, temporal pole, fusiform gyrus, and amygdalohippocampal transition zone*.

### Mouse Models of Neurodegenerative Disease Differentially Express Microglial Genes in Cortex and Hippocampus

Given that our analysis of healthy human brain revealed enriched expression of the microglial gene set in several regions affected early in the course of AD, we next explored the expression profile of microglial genes in several mouse models of AD and FTLD [(16); Mouseac project; www.mouseac.org]. Of the 30 human microglial genes identified in our prior analyses, 20 had data mapping to an orthologous mouse gene and passing quality control (as described above).

**Figure 2:**
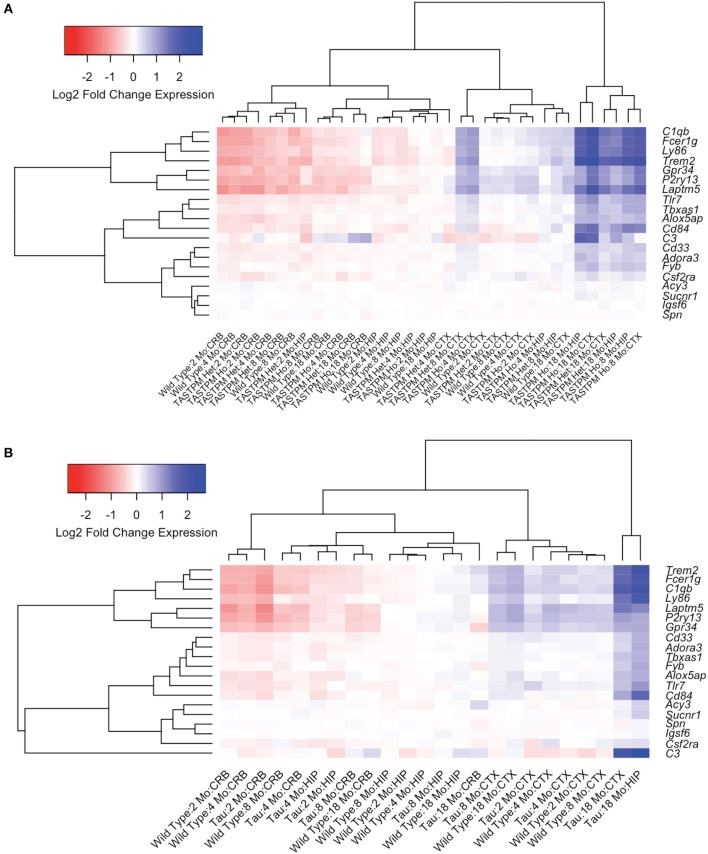
Microglial gene expression demonstrates age- and region-specific effects in neurodegenerative disease. To explore the temporal and spatial patterns of microglial gene expression in neurodegenerative disease, we used data from transgenic mouse models of Alzheimer's disease (AD; TASTPM mouse model) and tauopathy (Tau; P301L mouse model) from the Mouseac project (16). Hierarchical clustering analyses revealed that microglial gene expression is broadly divided into four groups, the first three of which are regionally-specific and attributable to whether the tissue sample was cerebellum, hippocampus, or cortex [shown toward the left in **(A,B)**]. The fourth group [shown farthest to the right in **(A,B)**] was driven by transgene status. In TASTPM mice, the fourth group exclusively included transgenic model tissue either heterozygous or homozygous for the TASTPM transgenes from either the hippocampus or cortex **(A)**. Further, the samples in the disease specific group tended to come from older mouse groupings, with all entries aged at least 8 months **(A)**. Data from the tau P301L mouse demonstrated congruent patterns when compared to the TASTPM mouse model **(B)**. CRB, Cerebellum; HIP, Hippocampus; CTX, Cortex; Mo, Month; AD, TASTPM mouse model; Tau, P301L tau mouse model; Het., Heterozygous; Ho., Homozygous.

We started by comparing the main effects of brain region, age, and transgene status. ANOVA analyses revealed markedly different microglial gene expression relative to wild type mice when compared by brain region and with increasing age. Using a strict Bonferroni correction for multiple testing (*p* = 0.000357), 16/20 microglial genes (80%) showed significant differences by brain region in the TASTPM mouse model data (including both homozygote and heterozygote mutation carriers, and accounting for gene dosage) combined with WT data (Table 5, Figure 2A) and 14/20 of microglial genes (70%) demonstrated significant differences in the P301L mouse model data combined with WT data (Table 6, Figure 2B). All 14 genes identified in the P301L mouse model overlapped with the genes identified in the TASTPM model. Similarly, the main effect of age was important in the combined TASTPM and WT mouse data as well as the P301L and WT mouse cohorts with 15/20 microglial genes (75%), inclusive of the ones observed in the other two models, showing differential expression (Tables 5, 6). Considering transgene status without regard to age or brain region (all ages and regions combined) showed that 15/20 microglial genes (75%) were significantly different in TASTPM (Table 5) and that 6/20 microglial genes (30%) were altered in P301L (Table 6). Five out of 6 genes identified in the P301L mouse model (*Cd33, Fcer1g, C1qb, C3*, and *Tlr7*) overlapped with genes upregulated in the TASTPM model.

**Table 5:** Microglial gene expression changes in the TASTPM mouse model.

**Gene**	**TASTPM**	**Age**	**Region**	**TASTPM:age**	**TASTPM:region**	**Age:region**	**TASTPM:age:region**
*Cd33*	**3.75E-23**	**1.43E-14**	**3.77E-36**	**5.62E-08**	**2.51E-05**	0.003	6.62E-04
*Cd84*	**5.34E-31**	**4.23E-25**	**9.24E-16**	**3.55E-14**	**1.97E-10**	**5.22E-06**	**5.80E-07**
*Fyb*	**1.73E-18**	**6.02E-16**	**1.21E-10**	**6.24E-06**	**3.11E-04**	**1.93E-05**	**9.05E-06**
*Fcer1g*	**6.39E-34**	**1.04E-25**	**3.47E-49**	**7.08E-11**	**1.47E-06**	0.106	**3.35E-05**
*Gpr34*	**2.26E-21**	**6.37E-18**	**3.42E-67**	**9.21E-09**	**1.43E-05**	0.016	**4.16E-05**
*Adora3*	**3.52E-13**	**4.56E-18**	**3.95E-24**	**9.84E-11**	**6.22E-06**	**1.37E-05**	**1.89E-07**
*Acy3*	0.071	0.494	0.010	0.772	8.30E-04	0.027	0.019
*Alox5ap*	**1.17E-15**	**5.69E-28**	**5.86E-32**	**6.58E-09**	**2.02E-04**	0.153	5.10E-04
*Csf2ra*	0.608	0.013	**1.31E-12**	0.090	0.004	0.984	0.639
*C1qb*	**1.29E-38**	**2.49E-29**	**6.95E-51**	**1.37E-10**	**1.87E-04**	0.198	**8.80E-06**
*C3*	**3.24E-16**	**9.05E-34**	**6.32E-06**	**2.53E-10**	0.290	0.003	**3.31E-04**
*Igsf6*	0.260	0.510	0.845	0.712	0.910	0.160	0.969
*Ly86*	**2.91E-29**	**3.04E-26**	**8.80E-33**	**2.74E-10**	**2.27E-07**	0.037	**2.93E-06**
*Laptm5*	**9.51E-21**	**9.32E-23**	**1.70E-60**	**8.07E-09**	**3.16E-05**	0.417	**2.19E-04**
*P2ry13*	**2.00E-15**	**1.82E-18**	**2.30E-74**	**2.99E-05**	0.005	0.177	0.001
*Spn*	0.137	0.518	0.648	0.593	0.723	0.746	0.698
*Sucnr1*	0.186	0.011	0.481	0.003	0.044	0.026	0.044
*Tbxas1*	**8.07E-29**	**1.77E-15**	**2.19E-21**	**2.35E-09**	**4.10E-10**	**1.35E-04**	**1.23E-04**
*Tlr7*	**6.24E-29**	**3.88E-17**	**8.02E-38**	**9.58E-12**	**2.89E-08**	**1.03E-04**	**1.22E-06**
*Trem2*	**1.20E-35**	**1.73E-30**	**3.46E-42**	**5.36E-14**	**5.45E-7**	**3.54E-04**	**2.11E-08**

*Analysis of variance (ANOVA) results from the TASTPM mouse model of Alzheimer's disease are shown when transgenic mice and wild type mice were compared by age and brain region (e.g., cerebellum, hippocampus, or cortex). Significant findings (Bonferroni p-value threshold of 0.000357) are shown in bold. Data are courtesy of the Mouseac project (16)*.

**Table 6:** Microglial gene expression changes in the P301L tau mouse model.

**Gene**	**P301L Tau**	**Age**	**Region**	**Tau:age**	**Tau:region**	**Age:region**	**Tau:age:region**
*Cd33*	**1.47E-04**	**1.89E-08**	**2.40E-24**	**4.55E-08**	0.178	0.029	**2.14E-07**
*Cd84*	0.002	**2.08E-11**	**1.54E-04**	**3.81E-09**	0.167	0.019	**1.28E-06**
*Fyb*	0.004	**2.96E-06**	**1.07E-04**	0.002	0.476	0.035	**3.72E-05**
*Fcer1g*	**2.92E-04**	**3.62E-18**	**5.55E-35**	**1.45E-12**	0.591	0.615	**2.38E-05**
*Gpr34*	0.483	**9.15E-08**	**1.89E-50**	**2.63E-04**	0.828	0.599	**2.21E-04**
*Adora3*	0.017	**2.17E-09**	**7.11E-14**	**6.78E-09**	0.307	0.016	**4.90E-07**
*Acy3*	**3.39E-04**	0.002	0.023	**1.73E-05**	0.011	0.660	0.362
*Alox5ap*	0.017	**2.39E-17**	**1.80E-18**	**8.91E-07**	0.793	0.291	**4.30E-06**
*Csf2ra*	0.018	0.046	0.003	0.015	0.408	0.851	0.130
*C1qb*	**1.81E-05**	**7.78E-19**	**6.21E-35**	**9.04E-11**	0.596	0.552	**2.38E-04**
*C3*	**5.94E-05**	**8.44E-15**	0.185	**2.28E-08**	0.086	0.005	**5.97E-05**
*Igsf6*	0.625	0.286	0.412	0.144	0.411	0.286	0.241
*Ly86*	0.007	**3.88E-15**	**2.87E-18**	**9.18E-08**	0.424	0.710	**2.35E-05**
*Laptm5*	0.207	**1.69E-10**	**5.48E-38**	**4.46E-05**	0.984	0.207	0.120
*P2ry13*	0.278	**1.16E-11**	**2.93E-55**	**5.36E-05**	0.566	0.358	0.060
*Spn*	0.750	0.278	0.466	0.277	0.996	0.564	0.101
*Sucnr1*	7.77E-04	0.005	0.697	**1.23E-05**	0.364	0.018	**4.95E-07**
*Tbxas1*	7.99E-04	**7.48E-09**	**4.48E-09**	**1.24E-08**	0.679	0.303	**2.88E-04**
*Tlr7*	**1.50E-04**	**2.50E-06**	**1.25E-19**	**2.38E-06**	0.810	0.151	**1.62E-04**
*Trem2*	**3.84E-4**	**2.15-19**	**2.76E-30**	**2.81E-11**	0.973	0.378	**1.97E-05**

*Analysis of variance (ANOVA) results from the P301L mouse model of tauopathy are shown when transgenic mice and wild type mice were compared by age and brain region (e.g., cerebellum, hippocampus, or cortex). Significant findings (Bonferroni p-value threshold of 0.000357) are shown in bold. Data are courtesy of the Mouseac project (16)*.

We next explored statistical interactions between age, brain region, and transgene status. In the TASTPM model, we observed significant interactions for transgene status by age (15/20), transgene status by region (13/20), and transgene status by age and region (12/20) (Table 5). Similar results were observed in the P301L model, with significant interactions for transgene status by age (16/20) and transgene status by age and region (14/20), but not transgene status by region (0/20) (Table 6). With respect to the transgene status by age analyses, the genes identified in each mouse model were almost identical, with the exceptions being *Fvb* in the TASTPM model and *Acy3* as well as *Sucnr1* in the P301L model. For the transgene status by age and region analyses, the two additional genes identified in the P301L tau model that were not identified in the TASTPM mouse were *Alox5ap* and *Cd33*—both of which were close to the significance cutoff (*p* = 0.000357).

**Figure 3:**
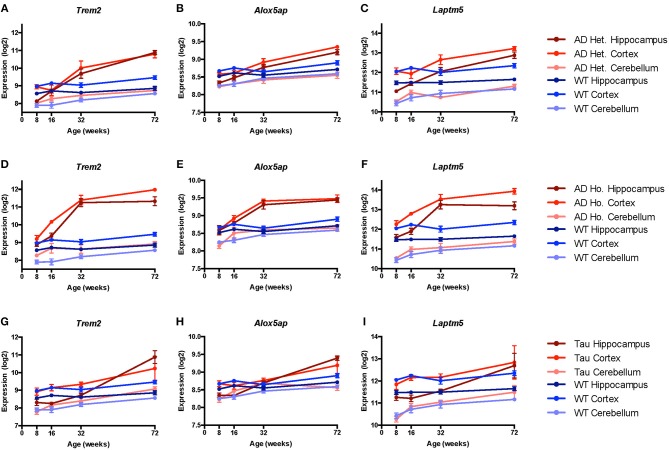
Microglial genes *Trem2, Alox5ap*, and *Laptm5* are upregulated in both pathologic and normal aging. Line plots depict selected microglial gene expression changes in the TASTPM (AD) and P301L transgenic mouse models of neurodegeneration relative to wild type (WT) mice across lifespan [data from www.mouseac.org (16)]. Expression profiles are shown for *Trem2* (encoding triggering receptor expressed on myeloid cells 2), *Alox5ap* (encoding arachidonate 5-lipoxygenase activating protein), and *Laptm5* (encoding lysosomal-associated protein transmembrane 5) that were differentially expressed over mouse lifespan when grouped by transgene status, tissue type, and age (*p*_raw_ < 0.05 by ANOVA). In both the TASTPM **(A–F)** and P301L **(G–I)** models, expression of microglial genes increased across the lifespan, especially for cortex and hippocampus with more modest changes in cerebellar tissue. Data for TASTPM heterozygotes and homozygotes was analyzed in one model (Table 4). For ease of comparison across multiple tissue types and conditions, expression data from TASTPM mice is shown for heterozygous **(A–C)** and homozygous **(D–F)** separately. Given this, gene expression ranges shown on the y-axis for *Trem2* **(A,D)**, *Alox5ap* **(B,E)**, and *Laptm5* **(C,F)** are the same. Data points show mean expression for each gene; error bars represent standard error of the mean. Het., Heterozygous; Ho., Homozygous.

Given the large number of genes implicated by our analyses, we chose three genes (*Trem2, Laptm5*, and *Alox5ap*) as exemplars of the expression patterns observed in the transgenic mice compared to controls at different ages (Figure 3). We chose *Trem2* given its known relevance to the pathobiology of AD and overall similarity to the upregulation patterns seen in other microglial genes (Figures 3A,D,G) (84, 85). Similarly, the expression profiles for *Alox5ap* (Figures 3B,E,H) and *Laptm5* (Figures 3C,F,I) were representative of the overall upregulation patterns seen in the microglial gene expression analyses. We chose *Alox5ap* and *Laptm5* to illustrate that expression profiles for the microglial gene module were similar irrespective of whether a gene has an established role in neurologic disease (e.g., *Trem2* and *Alox5ap*) or limited to no known role (e.g., *Laptm5*) in neurologic disease (Table 3). These illustrations corroborate the relationships shown in Figure 2 for other microglial genes (e.g., *Cd33, Cd84, Fyb, Fcer1g, Gpr34, Adora3, C1qb, C3, Ly86, P2ry13, Tbxas1*, and *Tlr7*), demonstrating that microglial gene expression is higher in diseased mice relative to wild-type and is upregulated in hippocampus and cortex compared to cerebellum. Finally, a steady increase in microglial gene expression with aging is evident in both wild-type and transgenic mice, but is especially prominent in the TASTPM and P301L mouse models of neurodegenerative disease. Given their purported role in disease-associated microglia [DAM; (86–88)], we provide additional plots for *Gpr34* and *P2ry13* in Figure S1.

### Microglia-Specific Genes Are Differentially Expressed in Human AD Tissue

We next characterized the expression of microglial genes in pathologically confirmed cases of AD relative to controls to test whether regions that show enriched microglial gene expression in the healthy brain are differentially impacted in AD. Data from the combined MSSM, Mayo, and ROSMAP studies amounted to 584 individuals with tissue samples from regions impacted early in AD (e.g., superior temporal cortex, parahippocampal gyrus, inferior frontal gyrus) and regions affected later in AD (e.g., cerebellum and frontal pole).

**Figure 4:**
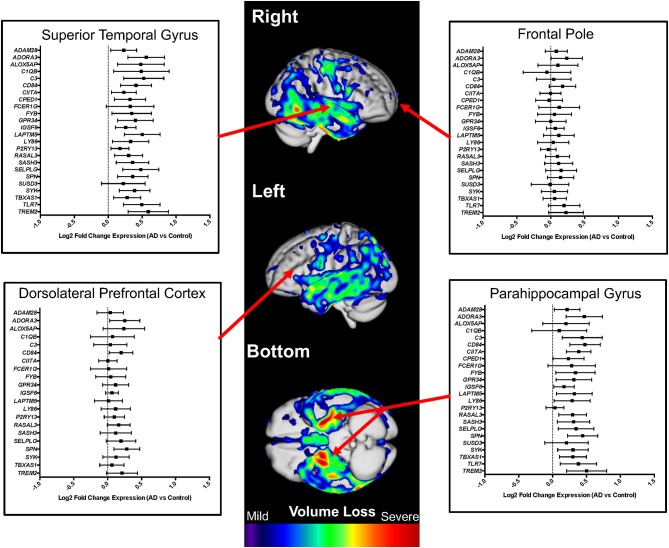
Microglial gene expression profiling in human Alzheimer's disease (AD) cases and controls. Differential expression analysis results from AD cases vs. controls are shown for multiple neuroanatomic regions on a representative atrophy map generated from AD cases and controls. Brain regions that demonstrate atrophy early in AD (e.g., superior temporal gyrus and parahippocampal gyrus) were also the regions with the highest expression of microglial genes. In contrast, regions that are generally spared until late in AD (e.g., frontal pole and dorsolateral prefrontal cortex) showed minimal to no differences in microglial gene expression. For the presented analyses, we evaluated the entire gene set when possible, omitting specific microglial genes only when expression data was not available. The atrophy map was generated using voxel-based morphometry and a sample of 60 clinically diagnosed AD cases compared to 60 normal controls. All individuals were seen at the UCSF Memory and Aging Center, scanned on a 3 Tesla scanner, and processed as previously described (26). The individuals used to generate a representative atrophy map were not used in the differential expression analyses.

Comparing expression in AD cases vs. controls, we found that microglial genes are highly upregulated, showing significant expression differences (*p* < 0.05) in some regions known to demonstrate significant atrophy in AD: superior temporal cortex (22 of 24 genes), parahippocampal gyrus (19 of 24 genes), inferior frontal gyrus (14 of 24 genes), and bulk temporal cortex (13 of 26 genes) (Figure 4, Table S1, Figure S2). Regions typically spared until very late in disease showed fewer significant (*p* < 0.05) differences: frontal pole (1 of 24 genes) and DLPFC (3 of 21 genes) (Figure 4, Table 1, Figure S2). Interestingly, the cerebellum showed mixed associations, with 13 of 26 genes upregulated and others showing no apparent changes in AD (Figure S2) despite being a region that is usually spared from atrophy in early AD.

We next explored whether microglial gene expression differed by sex amongst those diagnosed with AD. Our analyses suggested a subtle effect of increased expression of microglial genes in the regions most impacted in AD specific to females with significant (*p* < 0.05) upregulation in bulk temporal cortex (12 of 26 genes in females and 2 of 26 genes in males), superior temporal cortex (20 of 24 genes in females and 11 of 24 genes in males), and cerebellum (12 of 26 genes in females and 5 of 26 genes in males) (Figure S3). Of note, other structures implicated in our combined-sex AD analyses show similar expression profiles in both sexes like inferior frontal gyrus (8 of 24 genes in females and 10 of 24 genes in males) and parahippocampal gyrus (8 of 24 genes in females and 7 of 24 genes in males) (Figure S3, Table 1). Regions that did not demonstrate upregulation of microglial genes in AD such as frontal pole (4 out of 24 genes in females and 1 out of 24 genes in males) and DLPFC (0 out of 21 genes in females and 3 out of 21 genes in males) demonstrated minimal sex-specific effects in pathologically confirmed AD cases at *p* < 0.05.

### Microglia-Specific Genes Show AD-Specific Enrichment in Disease Risk

When generating a microglial gene expression profile using data-driven techniques, we hypothesized that the resulting microglial genes would be associated with risk for CNS immune disorders and, potentially, neurodegenerative disorders. Our gene expression analyses in mice and humans provide suggestive evidence implicating microglial genes in the pathophysiology of AD. However, they do not specifically test whether the identified microglial genes demonstrate enrichment for genes implicated in AD risk, which would implicate microglia at a deeper biological level and potentially suggest them as a selectively vulnerable cell type in AD. To address this question, we utilized an online platform analyzing over 2,500 GWAS publications and over 24,000 SNP trait associations (FUMA). Using a background dataset of 19,283 genes, we found that AD was, in fact, the only disease enriched (raw *p* = 8.87 × 10–6; FDR corrected *p* = 0.017; Figure S4; testing against over 3,000 unique diseases and traits) for the 30 microglial genes we identified using gene expression profiling in healthy controls.

## Discussion

### Summary of Findings

In this study we successfully identified 30 microglial genes with expression profiles significantly related to the well-established microglial marker, *TMEM119*. This microglial gene set was generated using *CellMapper*, a novel tool that enables the identification of networks of co-expressed genes. Analyzing this microglial gene expression module with finely dissected samples of healthy human brain, we identified 48 regions (25% of tested regions) showing significant enrichment for the microglial gene set, including particularly robust enrichment in a number of regions affected early in the course of AD (e.g., parahippocampal gyrus and inferior frontal gyrus). However, we also detected enrichment in regions that are not particularly affected in AD (e.g., frontal pole and DLPFC) which indicates that enrichment of microglial genes is not necessarily sufficient to predict region-specific vulnerability in AD.

Analysis of orthologous members of this microglial gene set in mouse models of AD and tauopathy revealed striking variation in expression level that was dictated by brain region, age, and transgene status, with highest expression observed in the aged cortex and hippocampus of mouse models of neurodegenerative disease. We then surveyed the microglial gene expression module in pathologically confirmed human AD cases and found striking upregulation of the microglial module in regions impacted early in the course of AD (e.g., superior temporal cortex, parahippocampal gyrus, and inferior frontal gyrus), and somewhat less robust upregulation in the cerebellum, which is largely unaffected in AD. Finally, we observed significant overlap between our microglial gene expression module and genes independently found to impart risk for AD.

Collectively, our findings demonstrate that microglial gene expression varies greatly by brain region and as a function of age, and responds dynamically to neurodegenerative processes in pathologically affected brain regions. Our findings for human AD are consistent with prior studies using independent datasets and modes of analysis which similarly implicated microglial and myeloid expression modules in AD risk and pathobiology (89, 90). Taken together, our findings and those cited above help explain why pathogenic mutations and rare variants in microglial genes may exert a disproportionately strong impact on risk for neurodegeneration.

### Study Limitations—Interpretation of Dynamic Gene Expression Networks

As with any study focusing on dynamic changes in gene expression networks, it is difficult to determine with certainty whether the measured changes in gene expression are a reflection of increases in microglial density (i.e., microglial “cellularity”), a reduction in the proportion of other cell types (e.g., loss of neurons due to ongoing neurodegeneration), or altered transcription of particular members of the microglial gene set [e.g., due to activation of a specific microglial gene expression program; for an in-depth discussion of these issues, see (91)]. These limitations are especially difficult to address when analyzing bulk tissue as was done in our study. A potential solution to the problems associated with microglial density is to analyze individual microglia using microdissection or single-cell RNA sequencing (scRNAseq; see below). Unfortunately, scRNAseq data have not, to our knowledge, been generated from finely dissected human brain regions demonstrating significant upregulation in this study. Such a dataset would enable important follow-up analyses that could provide additional insight into our current findings.

Microgliosis is a known feature of AD [reviewed in (92, 93)]; therefore, a relative increase in microglial density in pathologically affected regions is likely at least partially responsible for the microglial gene set upregulation we observed in human AD. This finding is further supported by evidence from AD cases demonstrating that microglial density is proportional to neurofibrillary tangle frequency and distribution (94). In addition, *TMEM119* has been shown to be stably and selectively expressed in adult microglia (9, 95), and appears to exhibit stable expression even in response to a variety of inflammatory conditions (9, 96). Taking all of the above into consideration, it would be tempting to speculate that the expression changes we observed in this study are primarily a reflection of microglial cellularity. Intriguingly, *P2ry13* and *Gpr34*, mouse orthologs of genes we identified in our human microglial gene set, are known to show down-regulation in so-called DAM (86–88) that are found in AD and other neurological disorders. *P2ry13* is an ADP receptor and part of the G-protein coupled receptor family—it is thought to play a role in hematopoiesis and immune function (97). The function and importance of *Gpr34* and its protein product remain largely undetermined, though its suspected role in detecting short chain fatty acids may implicate it in both metabolic and immune pathways (45, 98). That we observed *increased* expression of these genes (in both mouse and human) in bulk tissue from regions affected in neurodegeneration further indicates that the results we observe are driven, at least in part, by increased microglial cell density. Other potential explanations of the increased expression we observed include age differences at tissue collection and use of different AD mouse models.

On the other hand, *TREM2* is also a member of our gene expression module and is known to be upregulated in DAM (87, 99). Dynamic changes in our microglial gene expression module therefore likely reflect a combination of increases in microglial cellularity and activation of specific gene expression programs (e.g., microgliosis and concomitant activation of the DAM program). It should be mentioned here that our knowledge of the DAM program is currently limited primarily to what has been gleaned from mouse models. Thus, future studies are needed to elucidate human-specific aspects of DAM dynamics in neurodegeneration.

Our study relies heavily upon publicly available data from human and mouse studies of neurodegenerative disease and healthy aging. Strengths of this approach are that it enabled us to build cohorts of large sample size, detect subtle disease effects, and ensure replicability of our findings; using large public datasets is also a limitation because our analyses required pooling of samples from multiple cohorts (especially in the postmortem human tissue analyses). Combining cohorts from multiple studies requires detailed and careful correction for batch effects and covariate selection but was necessary to achieve adequate statistical power. Despite pooling data from multiple sources, we cannot fully disentangle whether our findings are specific to AD, due to microglial density patterns, or both. For instance, we do not have AD samples from globus pallidus or corpus callosum, which would enable us to test whether microglial genes showed increased expression as a function of AD in regions that show highly enriched microglial expression in healthy brain, but which are not impacted until very late in AD.

The mouse models we utilized harbor genetic mutations that cause familial AD and FTLD, and thus present a unique set of limitations. Familial neurodegenerative disease is rare and the pathological processes driving it may be substantially different than those driving sporadic disease (100). Further, mouse models often mimic only limited aspects of the corresponding human neuropathology. For instance, the TASTPM mouse model of AD has amyloid plaques and demonstrates memory deficits, but does not show neurofibrillary tangles (101).

The primary limitations of *CellMapper* include that it was designed to use microarray data (not yet validated using RNAseq data) and that it requires validated as well as highly specific cell markers to accurately generate a cellular expression profile. Although many of our secondary analyses utilized RNAseq data, we generated the microglial expression profile using microarray data from the Allen Brain Institute. We thus were not subject to the former limitation. Secondly, we utilized *TMEM119* as a marker of microglial expression, which was shown in 2016 to be a highly specific marker of brain-derived microglia (9). Prior to TMEM119's discovery as a marker of brain-derived microglia, we would have been hindered by the latter limitation.

### Microglia-Mediated Synapse Loss in AD and Autoimmune Disease

A major question in the field remains whether microglial activity in AD is largely beneficial or detrimental. Data from human genetics, particularly with respect to the hypomorphic R47H allele of *TREM2* that strongly increases risk for AD (32, 33, 102), provide strong evidence for a protective role for microglia. However, it is currently unknown whether the protective effect of “normally” functioning microglia (via sufficient TREM2 function) is a manifestation of normal microglial activity specifically during aging, over the entire lifespan, or perhaps even during development.

In contrast to developments in human genetics, many studies in mouse models of neurodegenerative disease have identified a harmful role for microglia, particularly as drivers of synapse loss [for example, (103)]. In particular, tauopathy has been suggested to be a driver of complement- and microglia-mediated synapse engulfment (104, 105). Strikingly, mechanistic dissections of the CNS manifestations of the autoimmune disorder, lupus, have demonstrated a role for type I interferon signaling and autoantibodies in promoting inappropriate complement- and microglia-mediated engulfment of synapses (5, 106). Thus, while microglia are clearly capable of protecting against neurodegeneration when functioning in a homeostatic manner, inappropriate activation may be sufficient to induce synapse loss and neurodegeneration. A critical area of future research will be to determine at which point(s) in aging and/or disease microglia are protective, and at which point (or in which specific contexts) their activities become harmful. A clear understanding of these issues will be critical in determining which microglial molecules and pathways to target for therapeutic intervention, and at which point in the course of disease.

### Future Directions: Single-Cell RNA Sequencing and Microglial Sexual Dimorphism

Several publications within the last 2 years have begun to employ scRNAseq and mass cytometry of microglia and other CNS immune cells as ways to understand (i) global alterations in immunological gene expression and (ii) dynamic changes in immune cell-type abundance as a function of brain anatomy, aging, and neurodegeneration (87, 95, 107, 108). The major advantage of these techniques is that they enable unbiased, global profiling of gene expression uninfluenced by changes in cellularity, as well as characterization of cell-type fluctuations as a function of anatomy, age, and disease. Given this, replication of our findings using scRNAseq technology will be critical to determine whether our findings were influenced primarily by microglial cellularity or by changes in microglial gene expression programs. Another critical issue for future work will be to determine the mechanisms responsible for sexually dimorphic microglial gene expression profiles reported in mice (28, 88) and to what extent they apply to human microglia. Our work comparing microglial gene expression in human brain tissue from AD cases suggests that the relationships observed in mice may apply to the human condition and is consistent with reports from humans showing that there is a sex dimorphism in non-diseased human tissue (109) and that microglial gene expression changes with aging (110–112). Our sex-specific findings, despite modest effect size, are a promising addition to this emerging literature, given that AD is one of the most common diseases of aging and shows sex-specific incidence and progression (113). As described above, extending our study in the future with the use of scRNAseq would likely provide deeper insight into sex- and age-specific effects on microglial gene expression changes in AD.

## Ethics Statement

This study was carried out in accordance with the recommendations of University of California, San Francisco Committee on Human Research. The protocol was approved by the University of California, San Francisco Committee on Human Research. All subjects gave written informed consent in accordance with the Declaration of Helsinki.

## Author Contributions

LB conceived the study, performed statistical analyses, produced the figures, and drafted the manuscript. DS provided feedback on analyses, produced the figures, and drafted the manuscript. JY conceived the study, supervised all study activities, and drafted the manuscript.

### Conflict of Interest Statement

The authors declare that the research was conducted in the absence of any commercial or financial relationships that could be construed as a potential conflict of interest.
