# Supplementary material for: The Transcriptional Landscape of Microglial Genes in Aging and Neurodegenerative Disease
Source: Front Immunol. 2019 Jun 4;10:1170. doi: 10.3389/fimmu.2019.01170 (PMC6557985; doi:10.3389/fimmu.2019.01170)
Supplement: Supplementary file 1 [file Table_1.pdf]

Table S1. Differential Expression Analyses

Analysis	Gene	Cohort	Tissue Type	Log Fold-Change	Lower CI	Upper CI	p-value
Diagnosis	*ALOX5AP*	MAYO	CBE	0.873	0.523	1.224	1.35E-06
Diagnosis	*TLR7*	MAYO	CBE	0.693	0.309	1.078	4.32E-04
Diagnosis	*C1QB*	MAYO	CBE	0.672	0.277	1.067	8.99E-04
Diagnosis	*FCER1G*	MAYO	CBE	0.593	0.258	0.928	0.001
Diagnosis	*FYB*	MAYO	CBE	0.544	0.202	0.886	0.002
Diagnosis	*CPED1*	MAYO	CBE	0.497	0.173	0.821	0.003
Diagnosis	*SASH3*	MAYO	CBE	0.435	0.132	0.738	0.005
Diagnosis	*SPN*	MAYO	CBE	0.417	0.116	0.717	0.007
Diagnosis	*CD84*	MAYO	CBE	0.481	0.124	0.838	0.008
Diagnosis	*IGSF6*	MAYO	CBE	0.265	0.056	0.474	0.013
Diagnosis	*LAPTM5*	MAYO	CBE	0.361	0.069	0.653	0.015
Diagnosis	*GPR34*	MAYO	CBE	0.462	0.087	0.837	0.016
Diagnosis	*TREM2*	MAYO	CBE	0.329	0.01	0.647	0.043
Diagnosis	*TBXAS1*	MAYO	CBE	0.22	-0.009	0.448	0.059
Diagnosis	*CIITA*	MAYO	CBE	0.18	-0.024	0.384	0.083
Diagnosis	*RASAL3*	MAYO	CBE	0.18	-0.046	0.406	0.119
Diagnosis	*C3*	MAYO	CBE	0.254	-0.075	0.584	0.13
Diagnosis	*ADORA3*	MAYO	CBE	0.215	-0.09	0.52	0.166
Diagnosis	*SYK*	MAYO	CBE	0.187	-0.085	0.459	0.178
Diagnosis	*SELPLG*	MAYO	CBE	0.141	-0.136	0.418	0.317
Diagnosis	*LY86*	MAYO	CBE	0.133	-0.156	0.423	0.366
Diagnosis	*CD33*	MAYO	CBE	0.119	-0.186	0.424	0.444
Diagnosis	*P2RY13*	MAYO	CBE	-0.054	-0.356	0.248	0.726
Diagnosis	*SUSD3*	MAYO	CBE	-0.052	-0.373	0.269	0.75
Diagnosis	*ADAM28*	MAYO	CBE	0.04	-0.262	0.341	0.796
Diagnosis	*ACY3*	MAYO	CBE	-0.053	-0.546	0.44	0.833

Diagnosis	*ALOX5AP*	MAYO	TCX	0.68	0.327	1.033	1.75E-04
Diagnosis	*FCER1G*	MAYO	TCX	0.641	0.302	0.979	2.26E-04
Diagnosis	*TREM2*	MAYO	TCX	0.579	0.257	0.9	4.39E-04
Diagnosis	*ADORA3*	MAYO	TCX	0.502	0.194	0.809	0.001
Diagnosis	*CPED1*	MAYO	TCX	0.507	0.181	0.833	0.002
Diagnosis	*C3*	MAYO	TCX	0.513	0.18	0.846	0.003
Diagnosis	*CD84*	MAYO	TCX	0.516	0.156	0.877	0.005
Diagnosis	*SASH3*	MAYO	TCX	0.429	0.124	0.734	0.006
Diagnosis	*C1QB*	MAYO	TCX	0.551	0.149	0.952	0.007
Diagnosis	*CIITA*	MAYO	TCX	0.274	0.069	0.48	0.009
Diagnosis	*SPN*	MAYO	TCX	0.36	0.056	0.663	0.02
Diagnosis	*FYB*	MAYO	TCX	0.388	0.043	0.733	0.028
Diagnosis	*GPR34*	MAYO	TCX	0.407	0.029	0.785	0.035
Diagnosis	*TLR7*	MAYO	TCX	0.354	-0.034	0.741	0.074
Diagnosis	*LAPTM5*	MAYO	TCX	0.268	-0.027	0.563	0.075
Diagnosis	*TBXAS1*	MAYO	TCX	0.16	-0.07	0.391	0.172
Diagnosis	*SYK*	MAYO	TCX	0.162	-0.112	0.437	0.247
Diagnosis	*P2RY13*	MAYO	TCX	-0.14	-0.444	0.164	0.366
Diagnosis	*LY86*	MAYO	TCX	0.124	-0.168	0.416	0.404
Diagnosis	*RASAL3*	MAYO	TCX	0.091	-0.137	0.319	0.432
Diagnosis	*CD33*	MAYO	TCX	0.121	-0.187	0.429	0.44
Diagnosis	*SUSD3*	MAYO	TCX	-0.08	-0.404	0.244	0.628
Diagnosis	*ACY3*	MAYO	TCX	0.081	-0.416	0.578	0.749
Diagnosis	*IGSF6*	MAYO	TCX	0.027	-0.183	0.238	0.8
Diagnosis	*ADAM28*	MAYO	TCX	-0.019	-0.323	0.285	0.902
Diagnosis	*SELPLG*	MAYO	TCX	-0.001	-0.28	0.278	0.995
Diagnosis	*ADORA3*	MSSM	FP	0.236	0.004	0.467	0.046
Diagnosis	*CD84*	MSSM	FP	0.174	-0.021	0.368	0.081
Diagnosis	*TREM2*	MSSM	FP	0.224	-0.032	0.48	0.086

Diagnosis	*TLR7*	MSSM	FP	0.196	-0.035	0.428	0.097
Diagnosis	*SPN*	MSSM	FP	0.146	-0.047	0.339	0.139
Diagnosis	*SELPLG*	MSSM	FP	0.153	-0.075	0.381	0.187
Diagnosis	*SASH3*	MSSM	FP	0.117	-0.09	0.323	0.268
Diagnosis	*RASAL3*	MSSM	FP	0.099	-0.077	0.276	0.27
Diagnosis	*LAPTM5*	MSSM	FP	0.12	-0.106	0.346	0.298
Diagnosis	*IGSF6*	MSSM	FP	0.067	-0.063	0.198	0.31
Diagnosis	*ADAM28*	MSSM	FP	0.082	-0.081	0.246	0.323
Diagnosis	*FCER1G*	MSSM	FP	0.125	-0.176	0.427	0.414
Diagnosis	*ALOX5AP*	MSSM	FP	0.106	-0.189	0.402	0.481
Diagnosis	*TBXAS1*	MSSM	FP	0.059	-0.113	0.23	0.502
Diagnosis	*P2RY13*	MSSM	FP	-0.035	-0.149	0.079	0.545
Diagnosis	*SYK*	MSSM	FP	0.054	-0.141	0.249	0.587
Diagnosis	*FYB*	MSSM	FP	0.056	-0.194	0.306	0.661
Diagnosis	*C3*	MSSM	FP	0.044	-0.211	0.298	0.736
Diagnosis	*C1QB*	MSSM	FP	-0.057	-0.408	0.294	0.75
Diagnosis	*LY86*	MSSM	FP	0.037	-0.194	0.268	0.753
Diagnosis	*CPED1*	MSSM	FP	-0.024	-0.222	0.174	0.811
Diagnosis	*SUSD3*	MSSM	FP	-0.009	-0.286	0.268	0.948
Diagnosis	*CIITA*	MSSM	FP	-0.003	-0.159	0.153	0.97
Diagnosis	*GPR34*	MSSM	FP	0	-0.226	0.226	0.999
Diagnosis	*SPN*	MSSM	IFG	0.491	0.274	0.709	1.10E-05
Diagnosis	*SELPLG*	MSSM	IFG	0.577	0.319	0.834	1.30E-05
Diagnosis	*CD84*	MSSM	IFG	0.399	0.179	0.619	3.97E-04
Diagnosis	*SASH3*	MSSM	IFG	0.354	0.121	0.587	0.003
Diagnosis	*ADORA3*	MSSM	IFG	0.393	0.132	0.655	0.003
Diagnosis	*IGSF6*	MSSM	IFG	0.212	0.065	0.36	0.005
Diagnosis	*GPR34*	MSSM	IFG	0.371	0.115	0.627	0.005
Diagnosis	*TREM2*	MSSM	IFG	0.392	0.102	0.681	0.008

Diagnosis	*TLR7*	MSSM	IFG	0.348	0.087	0.61	0.009
Diagnosis	*C3*	MSSM	IFG	0.363	0.075	0.651	0.014
Diagnosis	*FCER1G*	MSSM	IFG	0.417	0.077	0.758	0.016
Diagnosis	*RASAL3*	MSSM	IFG	0.238	0.038	0.437	0.02
Diagnosis	*SUSD3*	MSSM	IFG	0.355	0.043	0.668	0.026
Diagnosis	*SYK*	MSSM	IFG	0.245	0.025	0.465	0.029
Diagnosis	*LAPTM5*	MSSM	IFG	0.251	-0.005	0.507	0.054
Diagnosis	*CPED1*	MSSM	IFG	0.201	-0.023	0.425	0.078
Diagnosis	*P2RY13*	MSSM	IFG	0.115	-0.013	0.244	0.079
Diagnosis	*ALOX5AP*	MSSM	IFG	0.297	-0.037	0.631	0.081
Diagnosis	*ADAM28*	MSSM	IFG	0.154	-0.031	0.339	0.102
Diagnosis	*TBXAS1*	MSSM	IFG	0.152	-0.042	0.346	0.124
Diagnosis	*LY86*	MSSM	IFG	0.189	-0.072	0.45	0.156
Diagnosis	*CIITA*	MSSM	IFG	0.114	-0.062	0.29	0.205
Diagnosis	*FYB*	MSSM	IFG	0.16	-0.122	0.443	0.266
Diagnosis	*C1QB*	MSSM	IFG	0.143	-0.254	0.541	0.478
Diagnosis	*CIITA*	MSSM	PHG	0.384	0.204	0.565	3.21E-05
Diagnosis	*CD84*	MSSM	PHG	0.478	0.252	0.703	3.58E-05
Diagnosis	*SPN*	MSSM	PHG	0.443	0.22	0.666	1.08E-04
Diagnosis	*TREM2*	MSSM	PHG	0.502	0.206	0.798	0.001
Diagnosis	*ADORA3*	MSSM	PHG	0.467	0.199	0.734	0.001
Diagnosis	*TBXAS1*	MSSM	PHG	0.299	0.1	0.498	0.003
Diagnosis	*C3*	MSSM	PHG	0.436	0.141	0.73	0.004
Diagnosis	*RASAL3*	MSSM	PHG	0.294	0.089	0.499	0.005
Diagnosis	*TLR7*	MSSM	PHG	0.38	0.112	0.648	0.006
Diagnosis	*SYK*	MSSM	PHG	0.299	0.074	0.525	0.009
Diagnosis	*SELPLG*	MSSM	PHG	0.346	0.083	0.61	0.01
Diagnosis	*SASH3*	MSSM	PHG	0.308	0.069	0.547	0.011
Diagnosis	*LAPTM5*	MSSM	PHG	0.321	0.059	0.583	0.016

Diagnosis	*GPR34*	MSSM	PHG	0.313	0.052	0.575	0.019
Diagnosis	*FYB*	MSSM	PHG	0.337	0.047	0.626	0.023
Diagnosis	*ADAM28*	MSSM	PHG	0.213	0.024	0.403	0.027
Diagnosis	*IGSF6*	MSSM	PHG	0.167	0.017	0.318	0.03
Diagnosis	*LY86*	MSSM	PHG	0.287	0.02	0.554	0.035
Diagnosis	*CPED1*	MSSM	PHG	0.234	0.005	0.463	0.045
Diagnosis	*FCER1G*	MSSM	PHG	0.281	-0.068	0.63	0.114
Diagnosis	*SUSD3*	MSSM	PHG	0.205	-0.116	0.525	0.21
Diagnosis	*ALOX5AP*	MSSM	PHG	0.197	-0.145	0.539	0.259
Diagnosis	*P2RY13*	MSSM	PHG	0.034	-0.098	0.166	0.612
Diagnosis	*C1QB*	MSSM	PHG	0.097	-0.309	0.504	0.638
Diagnosis	*ADORA3*	MSSM	STG	0.562	0.292	0.831	4.79E-05
Diagnosis	*TREM2*	MSSM	STG	0.591	0.293	0.889	1.07E-04
Diagnosis	*LAPTM5*	MSSM	STG	0.503	0.241	0.766	1.82E-04
Diagnosis	*TLR7*	MSSM	STG	0.495	0.226	0.765	3.29E-04
Diagnosis	*SELPLG*	MSSM	STG	0.481	0.216	0.746	3.94E-04
Diagnosis	*CD84*	MSSM	STG	0.41	0.183	0.637	4.10E-04
Diagnosis	*SYK*	MSSM	STG	0.389	0.162	0.615	0.001
Diagnosis	*IGSF6*	MSSM	STG	0.258	0.107	0.409	0.001
Diagnosis	*C3*	MSSM	STG	0.525	0.228	0.821	0.001
Diagnosis	*SPN*	MSSM	STG	0.362	0.137	0.586	0.002
Diagnosis	*SASH3*	MSSM	STG	0.359	0.119	0.6	0.003
Diagnosis	*GPR34*	MSSM	STG	0.401	0.138	0.664	0.003
Diagnosis	*RASAL3*	MSSM	STG	0.301	0.096	0.507	0.004
Diagnosis	*TBXAS1*	MSSM	STG	0.28	0.081	0.48	0.006
Diagnosis	*CPED1*	MSSM	STG	0.322	0.091	0.552	0.006
Diagnosis	*ALOX5AP*	MSSM	STG	0.482	0.138	0.826	0.006
Diagnosis	*P2RY13*	MSSM	STG	0.174	0.041	0.306	0.01
Diagnosis	*CIITA*	MSSM	STG	0.232	0.05	0.413	0.012

Diagnosis	*LY86*	MSSM	STG	0.331	0.063	0.599	0.016
Diagnosis	*ADAM28*	MSSM	STG	0.229	0.039	0.419	0.018
Diagnosis	*C1QB*	MSSM	STG	0.489	0.081	0.897	0.019
Diagnosis	*FYB*	MSSM	STG	0.347	0.056	0.637	0.02
Diagnosis	*FCER1G*	MSSM	STG	0.324	-0.027	0.675	0.07
Diagnosis	*SUSD3*	MSSM	STG	0.222	-0.1	0.544	0.176
Diagnosis	*SPN*	ROSMAP	DLPFC	0.282	0.091	0.472	0.004
Diagnosis	*CD84*	ROSMAP	DLPFC	0.198	0.024	0.372	0.026
Diagnosis	*ADORA3*	ROSMAP	DLPFC	0.253	0.031	0.474	0.026
Diagnosis	*RASAL3*	ROSMAP	DLPFC	0.161	-0.007	0.33	0.06
Diagnosis	*TREM2*	ROSMAP	DLPFC	0.211	-0.018	0.439	0.07
Diagnosis	*SELPLG*	ROSMAP	DLPFC	0.197	-0.021	0.415	0.076
Diagnosis	*ALOX5AP*	ROSMAP	DLPFC	0.238	-0.067	0.543	0.125
Diagnosis	*IGSF6*	ROSMAP	DLPFC	0.064	-0.032	0.159	0.192
Diagnosis	*P2RY13*	ROSMAP	DLPFC	0.099	-0.051	0.248	0.196
Diagnosis	*SYK*	ROSMAP	DLPFC	0.123	-0.072	0.318	0.215
Diagnosis	*GPR34*	ROSMAP	DLPFC	0.118	-0.073	0.308	0.226
Diagnosis	*LY86*	ROSMAP	DLPFC	0.118	-0.097	0.334	0.282
Diagnosis	*SASH3*	ROSMAP	DLPFC	0.12	-0.111	0.35	0.308
Diagnosis	*TBXAS1*	ROSMAP	DLPFC	0.063	-0.116	0.242	0.489
Diagnosis	*C1QB*	ROSMAP	DLPFC	0.071	-0.251	0.393	0.665
Diagnosis	*FYB*	ROSMAP	DLPFC	0.045	-0.175	0.265	0.688
Diagnosis	*ADAM28*	ROSMAP	DLPFC	0.038	-0.158	0.233	0.705
Diagnosis	*C3*	ROSMAP	DLPFC	0.039	-0.205	0.284	0.752
Diagnosis	*FCER1G*	ROSMAP	DLPFC	0.016	-0.251	0.283	0.907
Diagnosis	*LAPTM5*	ROSMAP	DLPFC	0.012	-0.195	0.219	0.909
Diagnosis	*CIITA*	ROSMAP	DLPFC	0.003	-0.133	0.139	0.964
Diagnosis X Female	*ALOX5AP*	MAYO	CBE	0.895	0.453	1.337	7.93E-05

Diagnosis X Female	*C1QB*	MAYO	CBE	0.862	0.365	1.359	0.001
Diagnosis X Female	*FCER1G*	MAYO	CBE	0.648	0.227	1.069	0.003
Diagnosis X Female	*CPED1*	MAYO	CBE	0.59	0.183	0.996	0.005
Diagnosis X Female	*TLR7*	MAYO	CBE	0.673	0.192	1.154	0.006
Diagnosis X Female	*SASH3*	MAYO	CBE	0.478	0.097	0.858	0.014
Diagnosis X Female	*GPR34*	MAYO	CBE	0.571	0.1	1.042	0.018
Diagnosis X Female	*IGSF6*	MAYO	CBE	0.307	0.045	0.57	0.022
Diagnosis X Female	*FYB*	MAYO	CBE	0.485	0.055	0.916	0.027
Diagnosis X Female	*CD84*	MAYO	CBE	0.506	0.057	0.954	0.027
Diagnosis X Female	*TREM2*	MAYO	CBE	0.445	0.046	0.844	0.029
Diagnosis X Female	*LAPTM5*	MAYO	CBE	0.376	0.008	0.743	0.045
Diagnosis X Female	*SPN*	MAYO	CBE	0.361	-0.016	0.738	0.06
Diagnosis X Female	*TBXAS1*	MAYO	CBE	0.224	-0.064	0.512	0.127
Diagnosis X Female	*SYK*	MAYO	CBE	0.265	-0.076	0.607	0.128
Diagnosis X Female	*CIITA*	MAYO	CBE	0.188	-0.068	0.444	0.15

Diagnosis X Female	*ADORA3*	MAYO	CBE	0.269	-0.115	0.654	0.169
Diagnosis X Female	*C3*	MAYO	CBE	0.287	-0.128	0.702	0.175
Diagnosis X Female	*RASAL3*	MAYO	CBE	0.187	-0.097	0.471	0.197
Diagnosis X Female	*CD33*	MAYO	CBE	0.185	-0.197	0.566	0.342
Diagnosis X Female	*LY86*	MAYO	CBE	0.128	-0.236	0.492	0.49
Diagnosis X Female	*SELPLG*	MAYO	CBE	0.101	-0.248	0.449	0.571
Diagnosis X Female	*ACY3*	MAYO	CBE	-0.163	-0.785	0.46	0.607
Diagnosis X Female	*SUSD3*	MAYO	CBE	-0.05	-0.452	0.352	0.808
Diagnosis X Female	*ADAM28*	MAYO	CBE	-0.03	-0.41	0.35	0.876
Diagnosis X Female	*P2RY13*	MAYO	CBE	-0.015	-0.393	0.363	0.938
Diagnosis X Female	*ALOX5AP*	MAYO	TCX	0.878	0.435	1.322	1.15E-04
Diagnosis X Female	*TREM2*	MAYO	TCX	0.761	0.359	1.163	2.24E-04
Diagnosis X Female	*FCER1G*	MAYO	TCX	0.803	0.378	1.227	2.29E-04
Diagnosis X Female	*CPED1*	MAYO	TCX	0.739	0.330	1.147	4.19E-04
Diagnosis X Female	*ADORA3*	MAYO	TCX	0.626	0.239	1.013	0.002

Diagnosis X Female	*C1QB*	MAYO	TCX	0.743	0.238	1.248	0.004
Diagnosis X Female	*CD84*	MAYO	TCX	0.659	0.208	1.110	0.004
Diagnosis X Female	*GPR34*	MAYO	TCX	0.677	0.204	1.150	0.005
Diagnosis X Female	*C3*	MAYO	TCX	0.578	0.160	0.995	0.007
Diagnosis X Female	*SPN*	MAYO	TCX	0.521	0.142	0.900	0.007
Diagnosis X Female	*TLR7*	MAYO	TCX	0.646	0.162	1.130	0.009
Diagnosis X Female	*CIITA*	MAYO	TCX	0.342	0.085	0.599	0.009
Diagnosis X Female	*SASH3*	MAYO	TCX	0.495	0.112	0.878	0.011
Diagnosis X Female	*FYB*	MAYO	TCX	0.431	-0.002	0.864	0.051
Diagnosis X Female	*LAPTM5*	MAYO	TCX	0.325	-0.047	0.696	0.086
Diagnosis X Female	*LY86*	MAYO	TCX	0.298	-0.068	0.664	0.110
Diagnosis X Female	*TBXAS1*	MAYO	TCX	0.222	-0.068	0.512	0.133
Diagnosis X Female	*CD33*	MAYO	TCX	0.281	-0.102	0.665	0.150
Diagnosis X Female	*SYK*	MAYO	TCX	0.222	-0.122	0.566	0.205
Diagnosis X Female	*IGSF6*	MAYO	TCX	0.147	-0.117	0.411	0.275

Diagnosis X Female	*RASAL3*	MAYO	TCX	0.127	-0.159	0.413	0.382
Diagnosis X Female	*SELPLG*	MAYO	TCX	0.125	-0.226	0.475	0.486
Diagnosis X Female	*ACY3*	MAYO	TCX	0.176	-0.450	0.802	0.581
Diagnosis X Female	*SUSD3*	MAYO	TCX	0.102	-0.302	0.507	0.619
Diagnosis X Female	*P2RY13*	MAYO	TCX	-0.041	-0.422	0.339	0.831
Diagnosis X Female	*ADAM28*	MAYO	TCX	0.016	-0.366	0.398	0.934
Diagnosis X Female	*ADORA3*	MSSM	FP	0.337	0.034	0.641	0.029
Diagnosis X Female	*CD84*	MSSM	FP	0.28	0.025	0.535	0.032
Diagnosis X Female	*ADAM28*	MSSM	FP	0.224	0.01	0.438	0.04
Diagnosis X Female	*RASAL3*	MSSM	FP	0.241	0.01	0.473	0.041
Diagnosis X Female	*SELPLG*	MSSM	FP	0.286	-0.012	0.585	0.06
Diagnosis X Female	*SPN*	MSSM	FP	0.241	-0.012	0.494	0.062
Diagnosis X Female	*SYK*	MSSM	FP	0.24	-0.014	0.495	0.064
Diagnosis X Female	*TREM2*	MSSM	FP	0.309	-0.027	0.645	0.071
Diagnosis X Female	*SASH3*	MSSM	FP	0.236	-0.036	0.508	0.089

Diagnosis X Female	*CIITA*	MSSM	FP	0.175	-0.029	0.379	0.092
Diagnosis X Female	*TBXAS1*	MSSM	FP	0.176	-0.049	0.402	0.125
Diagnosis X Female	*TLR7*	MSSM	FP	0.204	-0.101	0.508	0.19
Diagnosis X Female	*LAPTM5*	MSSM	FP	0.192	-0.105	0.489	0.205
Diagnosis X Female	*SUSD3*	MSSM	FP	0.234	-0.129	0.597	0.206
Diagnosis X Female	*ALOX5AP*	MSSM	FP	0.232	-0.156	0.621	0.24
Diagnosis X Female	*C3*	MSSM	FP	0.163	-0.171	0.497	0.337
Diagnosis X Female	*IGSF6*	MSSM	FP	0.078	-0.093	0.249	0.371
Diagnosis X Female	*P2RY13*	MSSM	FP	-0.058	-0.207	0.091	0.446
Diagnosis X Female	*FYB*	MSSM	FP	0.091	-0.238	0.42	0.588
Diagnosis X Female	*LY86*	MSSM	FP	0.072	-0.232	0.375	0.643
Diagnosis X Female	*FCER1G*	MSSM	FP	0.092	-0.305	0.488	0.651
Diagnosis X Female	*GPR34*	MSSM	FP	0.055	-0.241	0.351	0.716
Diagnosis X Female	*C1QB*	MSSM	FP	-0.043	-0.505	0.419	0.856
Diagnosis X Female	*CPED1*	MSSM	FP	-0.001	-0.261	0.258	0.992

Diagnosis X Female	*SPN*	MSSM	IFG	0.525	0.235	0.816	4.06E-04
Diagnosis X Female	*SELPLG*	MSSM	IFG	0.581	0.238	0.924	0.001
Diagnosis X Female	*SASH3*	MSSM	IFG	0.41	0.098	0.722	0.01
Diagnosis X Female	*CD84*	MSSM	IFG	0.346	0.053	0.638	0.021
Diagnosis X Female	*ADORA3*	MSSM	IFG	0.408	0.06	0.756	0.022
Diagnosis X Female	*TREM2*	MSSM	IFG	0.394	0.008	0.78	0.045
Diagnosis X Female	*SYK*	MSSM	IFG	0.298	0.006	0.591	0.045
Diagnosis X Female	*FCER1G*	MSSM	IFG	0.465	0.01	0.92	0.045
Diagnosis X Female	*TLR7*	MSSM	IFG	0.343	-0.006	0.692	0.054
Diagnosis X Female	*ADAM28*	MSSM	IFG	0.211	-0.035	0.456	0.093
Diagnosis X Female	*LAPTM5*	MSSM	IFG	0.277	-0.065	0.618	0.112
Diagnosis X Female	*ALOX5AP*	MSSM	IFG	0.32	-0.126	0.766	0.16
Diagnosis X Female	*RASAL3*	MSSM	IFG	0.186	-0.079	0.452	0.168
Diagnosis X Female	*GPR34*	MSSM	IFG	0.236	-0.104	0.577	0.174
Diagnosis X Female	*IGSF6*	MSSM	IFG	0.134	-0.063	0.33	0.181

Diagnosis X Female	*CIITA*	MSSM	IFG	0.149	-0.085	0.383	0.211
Diagnosis X Female	*C3*	MSSM	IFG	0.233	-0.151	0.616	0.234
Diagnosis X Female	*SUSD3*	MSSM	IFG	0.232	-0.184	0.649	0.273
Diagnosis X Female	*P2RY13*	MSSM	IFG	0.086	-0.086	0.257	0.327
Diagnosis X Female	*TBXAS1*	MSSM	IFG	0.115	-0.144	0.373	0.385
Diagnosis X Female	*LY86*	MSSM	IFG	0.137	-0.212	0.485	0.442
Diagnosis X Female	*FYB*	MSSM	IFG	0.14	-0.238	0.517	0.468
Diagnosis X Female	*C1QB*	MSSM	IFG	0.153	-0.377	0.684	0.571
Diagnosis X Female	*CPED1*	MSSM	IFG	0.04	-0.257	0.338	0.791
Diagnosis X Female	*CIITA*	MSSM	PHG	0.445	0.21	0.679	2.14E-04
Diagnosis X Female	*CD84*	MSSM	PHG	0.387	0.093	0.68	0.01
Diagnosis X Female	*ADORA3*	MSSM	PHG	0.461	0.111	0.81	0.01
Diagnosis X Female	*SYK*	MSSM	PHG	0.348	0.055	0.641	0.02
Diagnosis X Female	*SPN*	MSSM	PHG	0.333	0.041	0.624	0.025
Diagnosis X Female	*ADAM28*	MSSM	PHG	0.266	0.019	0.512	0.034

Diagnosis X Female	*TBXAS1*	MSSM	PHG	0.275	0.015	0.535	0.038
Diagnosis X Female	*TREM2*	MSSM	PHG	0.39	0.003	0.777	0.048
Diagnosis X Female	*SASH3*	MSSM	PHG	0.284	-0.029	0.597	0.075
Diagnosis X Female	*IGSF6*	MSSM	PHG	0.173	-0.024	0.37	0.085
Diagnosis X Female	*TLR7*	MSSM	PHG	0.3	-0.051	0.651	0.093
Diagnosis X Female	*LAPTM5*	MSSM	PHG	0.284	-0.059	0.626	0.104
Diagnosis X Female	*RASAL3*	MSSM	PHG	0.218	-0.049	0.485	0.109
Diagnosis X Female	*C3*	MSSM	PHG	0.303	-0.082	0.687	0.122
Diagnosis X Female	*SELPLG*	MSSM	PHG	0.27	-0.073	0.614	0.123
Diagnosis X Female	*FYB*	MSSM	PHG	0.292	-0.087	0.67	0.131
Diagnosis X Female	*GPR34*	MSSM	PHG	0.25	-0.091	0.591	0.15
Diagnosis X Female	*LY86*	MSSM	PHG	0.237	-0.112	0.587	0.182
Diagnosis X Female	*FCER1G*	MSSM	PHG	0.305	-0.152	0.762	0.19
Diagnosis X Female	*P2RY13*	MSSM	PHG	0.077	-0.095	0.249	0.379
Diagnosis X Female	*C1QB*	MSSM	PHG	0.235	-0.297	0.767	0.386

Diagnosis X Female	*CPED1*	MSSM	PHG	0.127	-0.171	0.426	0.402
Diagnosis X Female	*ALOX5AP*	MSSM	PHG	0.157	-0.291	0.604	0.492
Diagnosis X Female	*SUSD3*	MSSM	PHG	0.132	-0.286	0.55	0.536
Diagnosis X Female	*ADORA3*	MSSM	STG	0.62	0.284	0.957	3.17E-04
Diagnosis X Female	*TREM2*	MSSM	STG	0.65	0.278	1.023	0.001
Diagnosis X Female	*SYK*	MSSM	STG	0.479	0.196	0.761	0.001
Diagnosis X Female	*LAPTM5*	MSSM	STG	0.565	0.236	0.894	0.001
Diagnosis X Female	*CD84*	MSSM	STG	0.414	0.131	0.697	0.004
Diagnosis X Female	*ADAM28*	MSSM	STG	0.341	0.103	0.578	0.005
Diagnosis X Female	*SELPLG*	MSSM	STG	0.469	0.138	0.8	0.006
Diagnosis X Female	*SASH3*	MSSM	STG	0.411	0.11	0.713	0.008
Diagnosis X Female	*TLR7*	MSSM	STG	0.437	0.099	0.775	0.011
Diagnosis X Female	*C3*	MSSM	STG	0.478	0.108	0.848	0.011
Diagnosis X Female	*TBXAS1*	MSSM	STG	0.311	0.061	0.561	0.015
Diagnosis X Female	*IGSF6*	MSSM	STG	0.226	0.037	0.416	0.019

Diagnosis X Female	*ALOX5AP*	MSSM	STG	0.515	0.085	0.946	0.019
Diagnosis X Female	*P2RY13*	MSSM	STG	0.186	0.021	0.352	0.028
Diagnosis X Female	*FYB*	MSSM	STG	0.408	0.043	0.772	0.028
Diagnosis X Female	*RASAL3*	MSSM	STG	0.281	0.024	0.537	0.032
Diagnosis X Female	*GPR34*	MSSM	STG	0.355	0.027	0.683	0.034
Diagnosis X Female	*SPN*	MSSM	STG	0.3	0.019	0.581	0.036
Diagnosis X Female	*C1QB*	MSSM	STG	0.517	0.006	1.029	0.048
Diagnosis X Female	*CIITA*	MSSM	STG	0.227	0.001	0.453	0.049
Diagnosis X Female	*LY86*	MSSM	STG	0.305	-0.031	0.642	0.075
Diagnosis X Female	*CPED1*	MSSM	STG	0.244	-0.044	0.532	0.096
Diagnosis X Female	*FCER1G*	MSSM	STG	0.338	-0.102	0.778	0.132
Diagnosis X Female	*SUSD3*	MSSM	STG	0.162	-0.241	0.564	0.431
Diagnosis X Female	*SPN*	ROSMAP	DLPFC	0.237	-0.004	0.478	0.054
Diagnosis X Female	*ADORA3*	ROSMAP	DLPFC	0.233	-0.048	0.514	0.104
Diagnosis X Female	*CD84*	ROSMAP	DLPFC	0.137	-0.082	0.357	0.22

Diagnosis X Female	*TREM2*	ROSMAP	DLPFC	0.166	-0.123	0.455	0.259
Diagnosis X Female	*C3*	ROSMAP	DLPFC	-0.173	-0.48	0.133	0.268
Diagnosis X Female	*CIITA*	ROSMAP	DLPFC	-0.082	-0.254	0.091	0.352
Diagnosis X Female	*C1QB*	ROSMAP	DLPFC	-0.18	-0.583	0.224	0.383
Diagnosis X Female	*FCER1G*	ROSMAP	DLPFC	-0.14	-0.476	0.195	0.411
Diagnosis X Female	*SELPLG*	ROSMAP	DLPFC	0.108	-0.167	0.384	0.44
Diagnosis X Female	*FYB*	ROSMAP	DLPFC	-0.104	-0.38	0.173	0.463
Diagnosis X Female	*RASAL3*	ROSMAP	DLPFC	0.072	-0.14	0.284	0.504
Diagnosis X Female	*P2RY13*	ROSMAP	DLPFC	0.064	-0.125	0.253	0.507
Diagnosis X Female	*IGSF6*	ROSMAP	DLPFC	0.039	-0.082	0.16	0.524
Diagnosis X Female	*LY86*	ROSMAP	DLPFC	-0.083	-0.353	0.188	0.549
Diagnosis X Female	*SYK*	ROSMAP	DLPFC	0.055	-0.191	0.301	0.661
Diagnosis X Female	*LAPTM5*	ROSMAP	DLPFC	-0.054	-0.316	0.208	0.686
Diagnosis X Female	*GPR34*	ROSMAP	DLPFC	0.034	-0.207	0.275	0.781
Diagnosis X Female	*ADAM28*	ROSMAP	DLPFC	-0.031	-0.278	0.215	0.803

Diagnosis X Female	*TBXAS1*	ROSMAP	DLPFC	-0.021	-0.247	0.204	0.853
Diagnosis X Female	*ALOX5AP*	ROSMAP	DLPFC	-0.029	-0.412	0.355	0.884
Diagnosis X Female	*SASH3*	ROSMAP	DLPFC	-0.001	-0.291	0.289	0.995
Diagnosis X Male	*ALOX5AP*	MAYO	CBE	0.828	0.335	1.32	0.001
Diagnosis X Male	*TLR7*	MAYO	CBE	0.716	0.179	1.253	0.009
Diagnosis X Male	*FYB*	MAYO	CBE	0.629	0.148	1.109	0.01
Diagnosis X Male	*SPN*	MAYO	CBE	0.486	0.065	0.906	0.024
Diagnosis X Male	*FCER1G*	MAYO	CBE	0.522	0.052	0.992	0.03
Diagnosis X Male	*SASH3*	MAYO	CBE	0.383	-0.042	0.808	0.077
Diagnosis X Male	*CD84*	MAYO	CBE	0.439	-0.062	0.94	0.086
Diagnosis X Male	*CPED1*	MAYO	CBE	0.385	-0.069	0.84	0.096
Diagnosis X Male	*LAPTM5*	MAYO	CBE	0.346	-0.064	0.757	0.098
Diagnosis X Male	*C1QB*	MAYO	CBE	0.422	-0.133	0.977	0.136
Diagnosis X Male	*IGSF6*	MAYO	CBE	0.202	-0.092	0.495	0.177
Diagnosis X Male	*TBXAS1*	MAYO	CBE	0.214	-0.107	0.535	0.191
Diagnosis X Male	*GPR34*	MAYO	CBE	0.306	-0.22	0.831	0.253
Diagnosis X Male	*CIITA*	MAYO	CBE	0.166	-0.12	0.452	0.255
Diagnosis X Male	*RASAL3*	MAYO	CBE	0.17	-0.148	0.487	0.295
Diagnosis X Male	*C3*	MAYO	CBE	0.209	-0.254	0.672	0.376
Diagnosis X Male	*SELPLG*	MAYO	CBE	0.17	-0.219	0.559	0.392
Diagnosis X Male	*TREM2*	MAYO	CBE	0.189	-0.257	0.634	0.406
Diagnosis X Male	*ADORA3*	MAYO	CBE	0.135	-0.294	0.564	0.537
Diagnosis X Male	*LY86*	MAYO	CBE	0.118	-0.289	0.525	0.569
Diagnosis X Male	*P2RY13*	MAYO	CBE	-0.118	-0.54	0.305	0.585
Diagnosis X Male	*ADAM28*	MAYO	CBE	0.113	-0.311	0.537	0.602
Diagnosis X Male	*SYK*	MAYO	CBE	0.075	-0.307	0.456	0.7

Diagnosis X Male	*SUSD3*	MAYO	CBE	-0.068	-0.516	0.381	0.767
Diagnosis X Male	*CD33*	MAYO	CBE	0.048	-0.378	0.474	0.826
Diagnosis X Male	*ACY3*	MAYO	CBE	0.062	-0.633	0.756	0.862
Diagnosis X Male	*C3*	MAYO	TCX	0.473	0.010	0.936	0.045
Diagnosis X Male	*FCER1G*	MAYO	TCX	0.472	0.001	0.944	0.050
Diagnosis X Male	*ALOX5AP*	MAYO	TCX	0.448	-0.045	0.941	0.075
Diagnosis X Male	*SASH3*	MAYO	TCX	0.368	-0.057	0.793	0.089
Diagnosis X Male	*ADORA3*	MAYO	TCX	0.359	-0.071	0.788	0.102
Diagnosis X Male	*TREM2*	MAYO	TCX	0.372	-0.075	0.818	0.102
Diagnosis X Male	*CD84*	MAYO	TCX	0.380	-0.120	0.881	0.136
Diagnosis X Male	*FYB*	MAYO	TCX	0.362	-0.118	0.842	0.139
Diagnosis X Male	*CIITA*	MAYO	TCX	0.208	-0.078	0.494	0.153
Diagnosis X Male	*SUSD3*	MAYO	TCX	-0.272	-0.721	0.177	0.235
Diagnosis X Male	*C1QB*	MAYO	TCX	0.334	-0.226	0.894	0.242
Diagnosis X Male	*LAPTM5*	MAYO	TCX	0.231	-0.181	0.643	0.271
Diagnosis X Male	*P2RY13*	MAYO	TCX	-0.229	-0.651	0.194	0.288
Diagnosis X Male	*CPED1*	MAYO	TCX	0.234	-0.220	0.688	0.311
Diagnosis X Male	*SPN*	MAYO	TCX	0.198	-0.223	0.619	0.355
Diagnosis X Male	*IGSF6*	MAYO	TCX	-0.110	-0.403	0.182	0.459
Diagnosis X Male	*SELPLG*	MAYO	TCX	-0.136	-0.525	0.254	0.494
Diagnosis X Male	*TBXAS1*	MAYO	TCX	0.096	-0.226	0.417	0.558
Diagnosis X Male	*SYK*	MAYO	TCX	0.112	-0.269	0.494	0.564
Diagnosis X Male	*RASAL3*	MAYO	TCX	0.064	-0.253	0.382	0.690
Diagnosis X Male	*LY86*	MAYO	TCX	-0.075	-0.481	0.332	0.718
Diagnosis X Male	*GPR34*	MAYO	TCX	0.087	-0.439	0.612	0.746
Diagnosis X Male	*CD33*	MAYO	TCX	-0.054	-0.480	0.372	0.804
Diagnosis X Male	*ADAM28*	MAYO	TCX	-0.031	-0.455	0.393	0.886
Diagnosis X Male	*ACY3*	MAYO	TCX	-0.032	-0.726	0.662	0.928
Diagnosis X Male	*TLR7*	MAYO	TCX	0.022	-0.515	0.560	0.935

Diagnosis X Male	*CIITA*	MSSM	FP	-0.268	-0.513	-0.024	0.032
Diagnosis X Male	*SUSD3*	MSSM	FP	-0.317	-0.753	0.119	0.153
Diagnosis X Male	*TLR7*	MSSM	FP	0.24	-0.126	0.606	0.198
Diagnosis X Male	*SYK*	MSSM	FP	-0.194	-0.5	0.111	0.213
Diagnosis X Male	*ADAM28*	MSSM	FP	-0.126	-0.384	0.131	0.334
Diagnosis X Male	*IGSF6*	MSSM	FP	0.081	-0.124	0.286	0.44
Diagnosis X Male	*RASAL3*	MSSM	FP	-0.105	-0.383	0.173	0.459
Diagnosis X Male	*TREM2*	MSSM	FP	0.15	-0.253	0.553	0.466
Diagnosis X Male	*FCER1G*	MSSM	FP	0.159	-0.318	0.635	0.513
Diagnosis X Male	*TBXAS1*	MSSM	FP	-0.085	-0.356	0.186	0.54
Diagnosis X Male	*ADORA3*	MSSM	FP	0.11	-0.254	0.475	0.553
Diagnosis X Male	*CD84*	MSSM	FP	0.077	-0.23	0.383	0.624
Diagnosis X Male	*SASH3*	MSSM	FP	-0.058	-0.384	0.269	0.729
Diagnosis X Male	*C3*	MSSM	FP	-0.068	-0.468	0.333	0.74
Diagnosis X Male	*CPED1*	MSSM	FP	-0.051	-0.363	0.26	0.747
Diagnosis X Male	*ALOX5AP*	MSSM	FP	-0.058	-0.524	0.408	0.807
Diagnosis X Male	*LAPTM5*	MSSM	FP	0.043	-0.313	0.4	0.812
Diagnosis X Male	*FYB*	MSSM	FP	0.041	-0.354	0.435	0.84
Diagnosis X Male	*C1QB*	MSSM	FP	-0.037	-0.591	0.517	0.896
Diagnosis X Male	*SPN*	MSSM	FP	0.019	-0.285	0.324	0.901
Diagnosis X Male	*P2RY13*	MSSM	FP	0.006	-0.174	0.185	0.951
Diagnosis X Male	*LY86*	MSSM	FP	0.009	-0.355	0.374	0.959
Diagnosis X Male	*GPR34*	MSSM	FP	-0.001	-0.357	0.354	0.995
Diagnosis X Male	*SELPLG*	MSSM	FP	0.001	-0.357	0.359	0.996
Diagnosis X Male	*SELPLG*	MSSM	IFG	0.665	0.268	1.061	0.001
Diagnosis X Male	*GPR34*	MSSM	IFG	0.6	0.207	0.993	0.003
Diagnosis X Male	*CD84*	MSSM	IFG	0.518	0.18	0.855	0.003
Diagnosis X Male	*SPN*	MSSM	IFG	0.491	0.156	0.826	0.004
Diagnosis X Male	*IGSF6*	MSSM	IFG	0.287	0.06	0.514	0.013

Diagnosis X Male	*CPED1*	MSSM	IFG	0.429	0.085	0.772	0.014
Diagnosis X Male	*ADORA3*	MSSM	IFG	0.46	0.058	0.862	0.025
Diagnosis X Male	*RASAL3*	MSSM	IFG	0.349	0.042	0.655	0.026
Diagnosis X Male	*SUSD3*	MSSM	IFG	0.534	0.054	1.015	0.029
Diagnosis X Male	*C3*	MSSM	IFG	0.459	0.015	0.902	0.043
Diagnosis X Male	*TLR7*	MSSM	IFG	0.377	-0.026	0.781	0.067
Diagnosis X Male	*TREM2*	MSSM	IFG	0.41	-0.036	0.856	0.071
Diagnosis X Male	*SASH3*	MSSM	IFG	0.292	-0.068	0.652	0.112
Diagnosis X Male	*P2RY13*	MSSM	IFG	0.147	-0.051	0.345	0.146
Diagnosis X Male	*TBXAS1*	MSSM	IFG	0.215	-0.084	0.514	0.158
Diagnosis X Male	*FCER1G*	MSSM	IFG	0.335	-0.191	0.861	0.211
Diagnosis X Male	*SYK*	MSSM	IFG	0.181	-0.157	0.518	0.294
Diagnosis X Male	*ALOX5AP*	MSSM	IFG	0.274	-0.242	0.789	0.297
Diagnosis X Male	*LAPTM5*	MSSM	IFG	0.201	-0.194	0.596	0.318
Diagnosis X Male	*LY86*	MSSM	IFG	0.2	-0.202	0.602	0.328
Diagnosis X Male	*FYB*	MSSM	IFG	0.161	-0.275	0.597	0.47
Diagnosis X Male	*ADAM28*	MSSM	IFG	0.081	-0.203	0.364	0.576
Diagnosis X Male	*CIITA*	MSSM	IFG	0.056	-0.214	0.326	0.683
Diagnosis X Male	*C1QB*	MSSM	IFG	0.071	-0.542	0.684	0.82
Diagnosis X Male	*SPN*	MSSM	PHG	0.536	0.182	0.889	0.003
Diagnosis X Male	*CD84*	MSSM	PHG	0.527	0.171	0.883	0.004
Diagnosis X Male	*TREM2*	MSSM	PHG	0.594	0.125	1.063	0.013
Diagnosis X Male	*C3*	MSSM	PHG	0.502	0.036	0.968	0.035
Diagnosis X Male	*TLR7*	MSSM	PHG	0.438	0.013	0.863	0.044
Diagnosis X Male	*RASAL3*	MSSM	PHG	0.326	0.003	0.649	0.048
Diagnosis X Male	*ADORA3*	MSSM	PHG	0.424	0	0.847	0.05
Diagnosis X Male	*SELPLG*	MSSM	PHG	0.413	-0.003	0.83	0.052
Diagnosis X Male	*TBXAS1*	MSSM	PHG	0.301	-0.014	0.615	0.061
Diagnosis X Male	*CPED1*	MSSM	PHG	0.334	-0.028	0.696	0.071

Diagnosis X Male	*CIITA*	MSSM	PHG	0.234	-0.051	0.518	0.107
Diagnosis X Male	*SASH3*	MSSM	PHG	0.293	-0.086	0.673	0.13
Diagnosis X Male	*GPR34*	MSSM	PHG	0.315	-0.098	0.728	0.135
Diagnosis X Male	*LY86*	MSSM	PHG	0.32	-0.103	0.744	0.138
Diagnosis X Male	*FYB*	MSSM	PHG	0.347	-0.112	0.806	0.138
Diagnosis X Male	*LAPTM5*	MSSM	PHG	0.25	-0.164	0.665	0.236
Diagnosis X Male	*IGSF6*	MSSM	PHG	0.13	-0.108	0.369	0.284
Diagnosis X Male	*SUSD3*	MSSM	PHG	0.236	-0.271	0.743	0.36
Diagnosis X Male	*P2RY13*	MSSM	PHG	-0.086	-0.294	0.123	0.418
Diagnosis X Male	*SYK*	MSSM	PHG	0.137	-0.218	0.492	0.449
Diagnosis X Male	*FCER1G*	MSSM	PHG	0.209	-0.344	0.763	0.458
Diagnosis X Male	*ALOX5AP*	MSSM	PHG	0.178	-0.364	0.72	0.52
Diagnosis X Male	*C1QB*	MSSM	PHG	-0.161	-0.805	0.484	0.625
Diagnosis X Male	*ADAM28*	MSSM	PHG	0.038	-0.261	0.337	0.804
Diagnosis X Male	TLR7	MSSM	STG	0.607	0.196	1.018	0.004
Diagnosis X Male	SPN	MSSM	STG	0.482	0.141	0.824	0.006
Diagnosis X Male	IGSF6	MSSM	STG	0.320	0.090	0.550	0.007
Diagnosis X Male	C3	MSSM	STG	0.607	0.156	1.059	0.008
Diagnosis X Male	CPED1	MSSM	STG	0.442	0.092	0.793	0.013
Diagnosis X Male	GPR34	MSSM	STG	0.498	0.098	0.898	0.015
Diagnosis X Male	SELPLG	MSSM	STG	0.500	0.096	0.903	0.015
Diagnosis X Male	CD84	MSSM	STG	0.427	0.082	0.772	0.015
Diagnosis X Male	ADORA3	MSSM	STG	0.489	0.079	0.900	0.020
Diagnosis X Male	TREM2	MSSM	STG	0.532	0.078	0.986	0.022
Diagnosis X Male	RASAL3	MSSM	STG	0.329	0.017	0.642	0.039
Diagnosis X Male	LY86	MSSM	STG	0.407	-0.003	0.816	0.051
Diagnosis X Male	LAPTM5	MSSM	STG	0.391	-0.009	0.792	0.056
Diagnosis X Male	ALOX5AP	MSSM	STG	0.435	-0.089	0.960	0.104
Diagnosis X Male	CIITA	MSSM	STG	0.218	-0.057	0.493	0.120

Diagnosis X Male	TBXAS1	MSSM	STG	0.238	-0.066	0.543	0.125
Diagnosis X Male	SASH3	MSSM	STG	0.280	-0.087	0.647	0.134
Diagnosis X Male	P2RY13	MSSM	STG	0.151	-0.051	0.353	0.142
Diagnosis X Male	C1QB	MSSM	STG	0.464	-0.159	1.088	0.144
Diagnosis X Male	SYK	MSSM	STG	0.229	-0.115	0.573	0.191
Diagnosis X Male	FCER1G	MSSM	STG	0.346	-0.190	0.881	0.206
Diagnosis X Male	SUSD3	MSSM	STG	0.307	-0.184	0.798	0.220
Diagnosis X Male	FYB	MSSM	STG	0.272	-0.172	0.716	0.229
Diagnosis X Male	ADAM28	MSSM	STG	0.039	-0.249	0.328	0.789
Diagnosis X Male	*ALOX5AP*	ROSMAP	DLPFC	0.594	0.119	1.069	0.014
Diagnosis X Male	*SPN*	ROSMAP	DLPFC	0.357	0.059	0.655	0.019
Diagnosis X Male	*LY86*	ROSMAP	DLPFC	0.356	0.021	0.691	0.037
Diagnosis X Male	*RASAL3*	ROSMAP	DLPFC	0.26	-0.002	0.522	0.052
Diagnosis X Male	*CD84*	ROSMAP	DLPFC	0.252	-0.02	0.523	0.069
Diagnosis X Male	*SELPLG*	ROSMAP	DLPFC	0.289	-0.051	0.629	0.096
Diagnosis X Male	*C3*	ROSMAP	DLPFC	0.297	-0.082	0.676	0.125
Diagnosis X Male	*SASH3*	ROSMAP	DLPFC	0.254	-0.105	0.613	0.166
Diagnosis X Male	*SYK*	ROSMAP	DLPFC	0.212	-0.092	0.516	0.172
Diagnosis X Male	*ADORA3*	ROSMAP	DLPFC	0.242	-0.105	0.588	0.172
Diagnosis X Male	*C1QB*	ROSMAP	DLPFC	0.345	-0.154	0.844	0.175
Diagnosis X Male	*GPR34*	ROSMAP	DLPFC	0.202	-0.095	0.499	0.183
Diagnosis X Male	*FYB*	ROSMAP	DLPFC	0.224	-0.118	0.567	0.198
Diagnosis X Male	*TREM2*	ROSMAP	DLPFC	0.234	-0.123	0.591	0.199
Diagnosis X Male	*CIITA*	ROSMAP	DLPFC	0.131	-0.082	0.344	0.227
Diagnosis X Male	*IGSF6*	ROSMAP	DLPFC	0.089	-0.061	0.239	0.245
Diagnosis X Male	*P2RY13*	ROSMAP	DLPFC	0.129	-0.105	0.363	0.28
Diagnosis X Male	*TBXAS1*	ROSMAP	DLPFC	0.128	-0.151	0.406	0.368
Diagnosis X Male	*FCER1G*	ROSMAP	DLPFC	0.179	-0.236	0.594	0.397
Diagnosis X Male	*ADAM28*	ROSMAP	DLPFC	0.086	-0.219	0.39	0.58

Diagnosis X Male	*LAPTM5*	ROSMAP	DLPFC	0.085	-0.238	0.409	0.604

Table S1. Expanded results corresponding to the plots shown in Figure 4 as well as Supplementary Figures 1 and 2 are provided for reference. All analyses are the results of linear models performed using the voom-limma package in R. Additional analytical details can be found at www.synapse.org under the study's identifier (syn14237651). The main effect of diagnosis (i.e. Alzheimer's disease vs control) is provided first. The secondary analyses testing for an interaction between diagnosis and sex are provided beneath the primary analyses. All p-values provided are raw. Mayo – Mayo Clinic Brain Bank Data (see syn14237651 for additional details); ROSMAP – Religious Orders Study and Memory and Aging Project study (see syn14237651 for additional details); MSSM – Mount Sinai Brain Bank study (see syn14237651 for additional details); CBE – Cerebellum; IFG – Inferior Frontal Gyrus; FP – Frontal Pole; PHG – Parahippocampal Gyrus; DLPFC – Dorsolateral Prefrontal Cortex; STG – Superior Temporal Gyrus; CI – Confidence Interval.
