# Supplementary material for: The Transcriptional Landscape of Microglial Genes in Aging and Neurodegenerative Disease
Source: Front Immunol. 2019 Jun 4;10:1170. doi: 10.3389/fimmu.2019.01170 (PMC6557985; doi:10.3389/fimmu.2019.01170)
Supplement: Supplementary file 2 [file Image_1.pdf]

Figure S1. *P2ry13* and *Gpr34* upregulation in both pathologic and normal aging

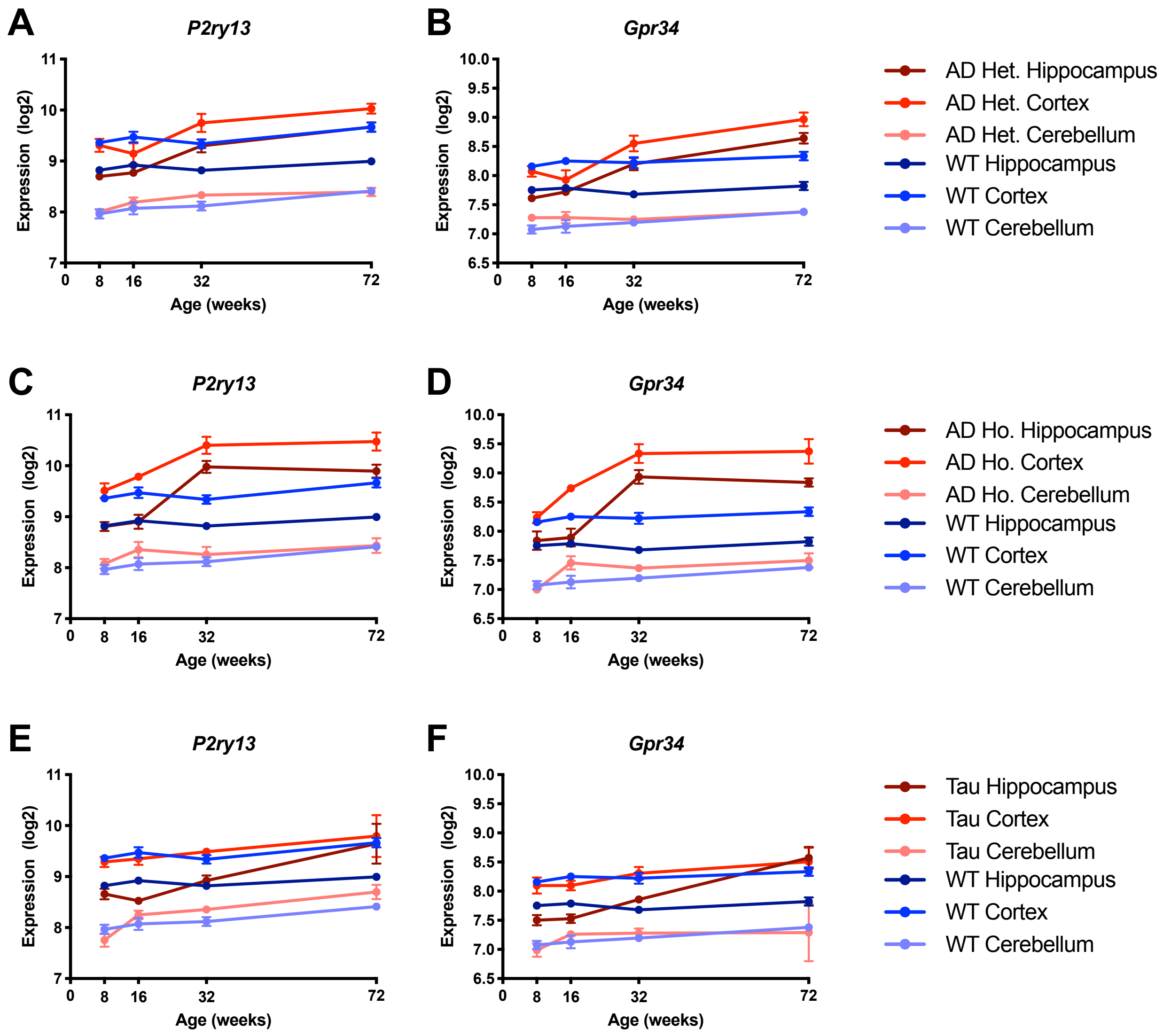


Figure S1. Line plots depict selected microglial gene expression changes in the TASTPM and P301L transgenic mouse models of neurodegeneration relative to wild type (WT) mice across lifespan (data from www.mouseac.org (Matarin et al., 2015)). Expression profiles are shown for *P2ry13* (encoding purinergic receptor P2Y13, G-protein coupled) and *Gpr34* (encoding G-protein coupled receptor 34) that were differentially expressed over mouse lifespan when grouped by transgene status, tissue type, and age ($p_{raw} < 0.05$ by ANOVA). In both the TASTPM (A-D) and P301L (E-F) models, expression of microglial genes increased across the lifespan, especially for cortex and hippocampus with more modest changes in cerebellar tissue. Data for TASTPM heterozygotes and homozygotes was analyzed in one model (Table 4). For ease of comparison across multiple tissue types and conditions, expression data from TASTPM mice is shown for heterozygous (A-B) and homozygous (C-D) separately. The axes for each gene are set to the same range to facilitate comparisons. Data points show mean expression for each gene; error bars represent standard error of the mean. Het. – Heterozygous; Ho – Homozygous.
