# Supplementary material for: The Transcriptional Landscape of Microglial Genes in Aging and Neurodegenerative Disease
Source: Front Immunol. 2019 Jun 4;10:1170. doi: 10.3389/fimmu.2019.01170 (PMC6557985; doi:10.3389/fimmu.2019.01170)
Supplement: Supplementary file 3 [file Image_2.pdf]

Figure S2. Microglial gene expression profiling in human AD cases and controls

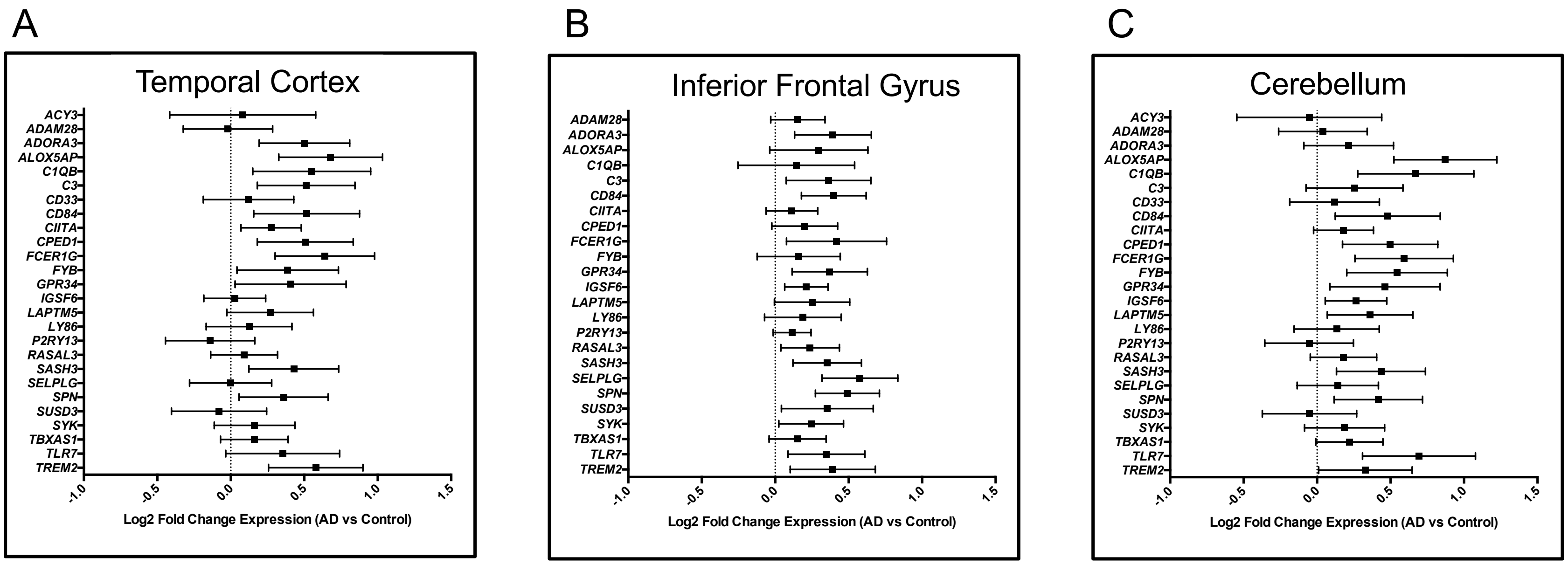

Figure S2 . Differential expression analysis results from Alzheimer’s Disease (AD) cases versus controls are shown for additional regions not shown in Figure 4. For the presented analyses, we evaluated the entire gene set when possible, omitting specific microglial genes only when expression data was not available.
