# Supplementary material for: The Transcriptional Landscape of Microglial Genes in Aging and Neurodegenerative Disease
Source: Front Immunol. 2019 Jun 4;10:1170. doi: 10.3389/fimmu.2019.01170 (PMC6557985; doi:10.3389/fimmu.2019.01170)
Supplement: Supplementary file 4 [file Image_3.pdf]

Figure S3. Microglial gene expression compared by sex in human AD cases and controls

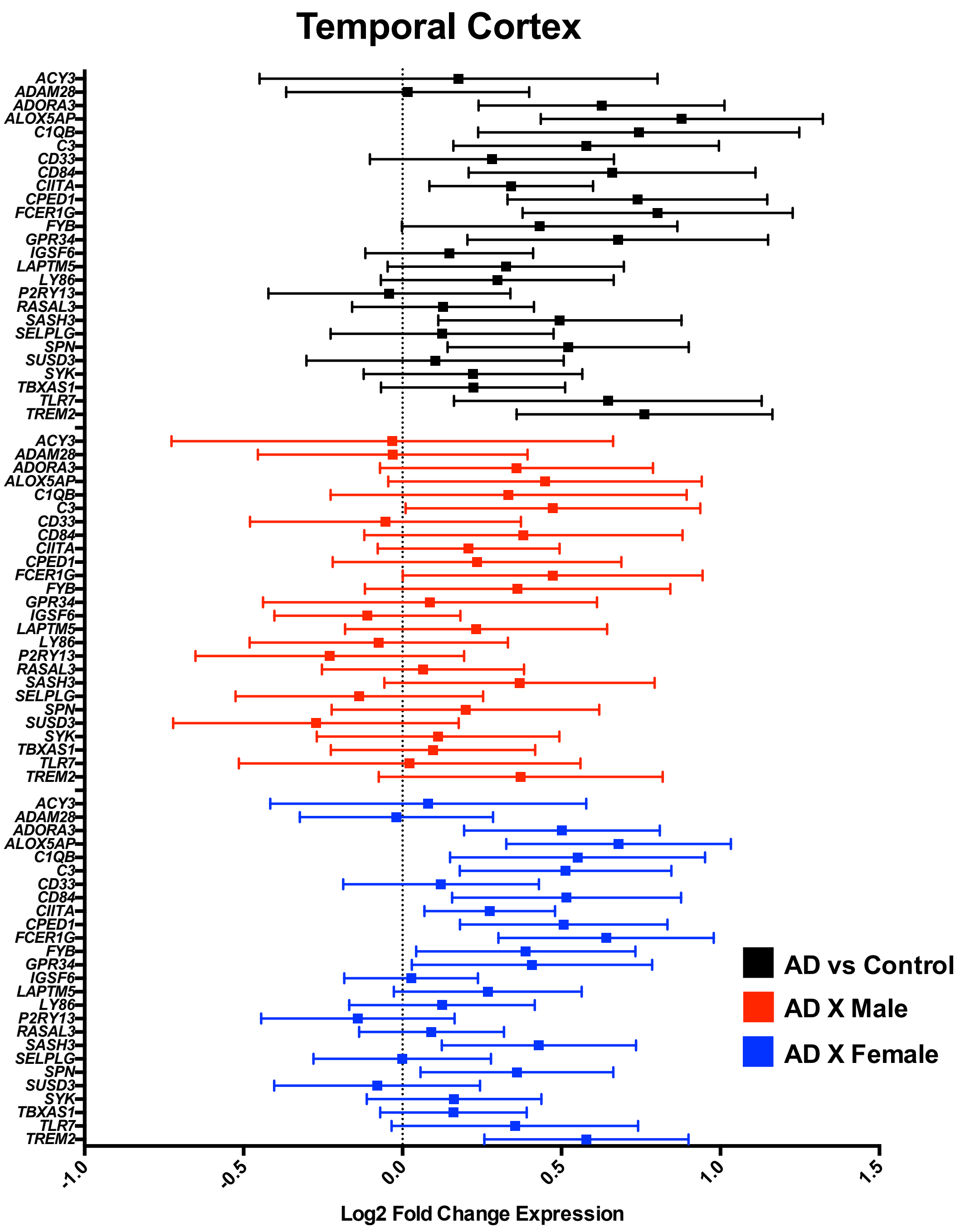

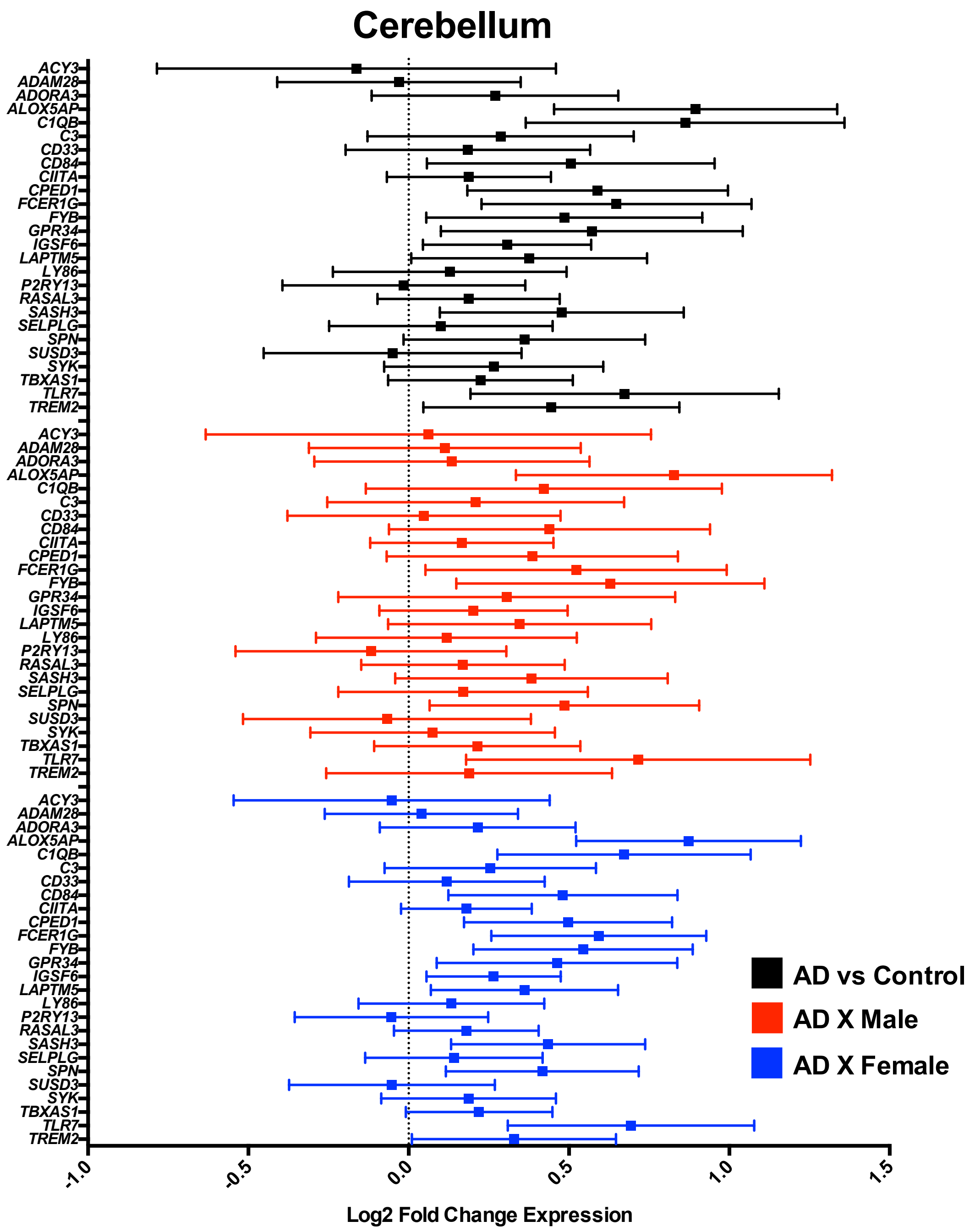
Cerebellum
ACY3
ADAM28
ADORA3
ALOX5AP
C1QB
C3
CD33
CD84
CIITA
CPED1
FCER1G
FYB
GPR34
IGSF6
LAPTM5
LY86
P2RY13
RASAL3
SASH3
SELPLG
SPN
SUSD3
SYK
TBXAS1
TLR7
TREM2
-1.0
-0.5
0.0
0.5
1.0
1.5
Log2 Fold Change Expression
AD vs Control
AD X Male
AD X Female

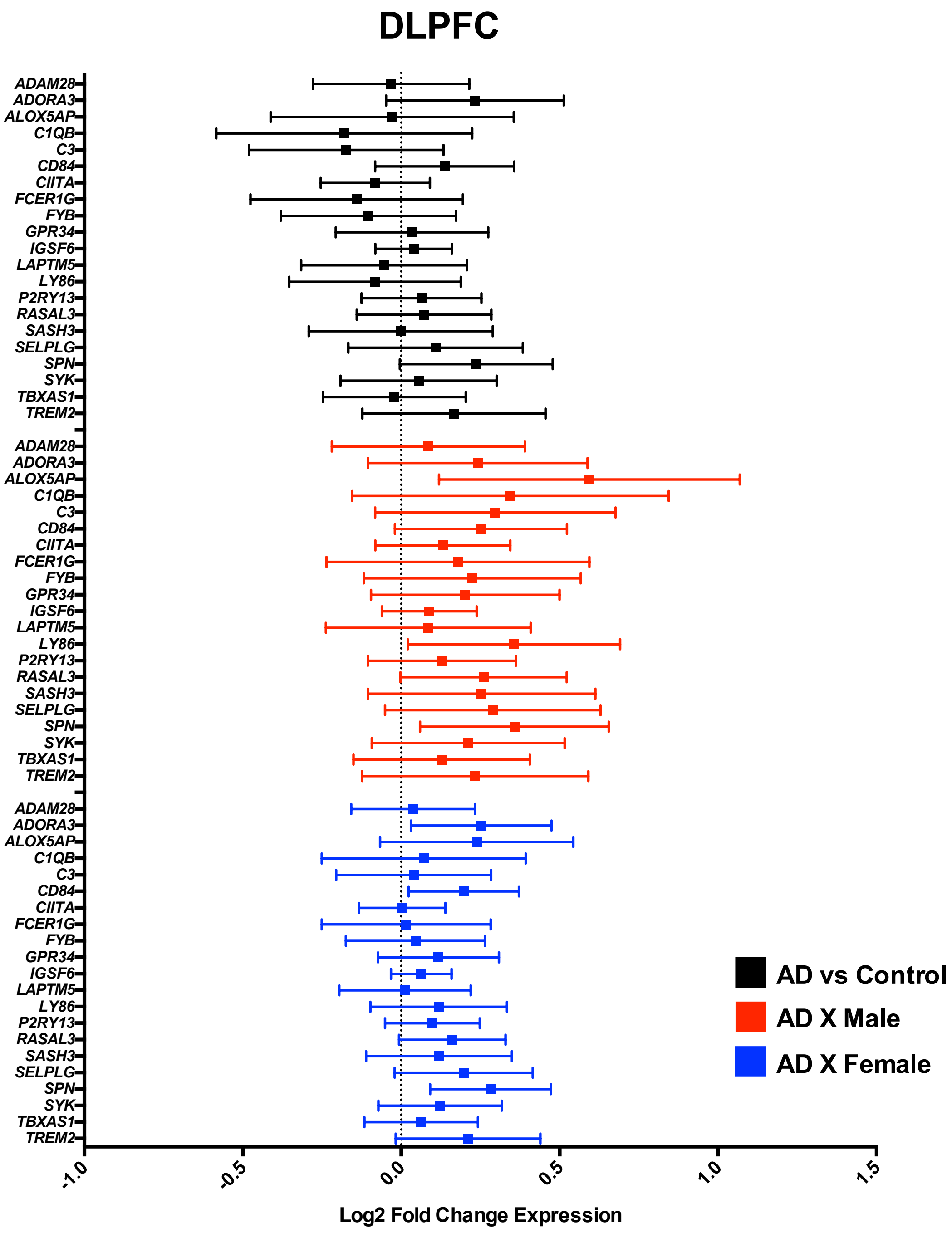
DLPFC
ADAM28
ADORA3
ALOX5AP
C1QB
C3
CD84
CIITA
FCER1G
FYB
GPR34
IGSF6
LAPTM5
LY86
P2RY13
RASAL3
SASH3
SELPLG
SPN
SYK
TBXAS1
TREM2
-1.0
-0.5
0.0
0.5
1.0
1.5
Log2 Fold Change Expression
AD vs Control
AD X Male
AD X Female

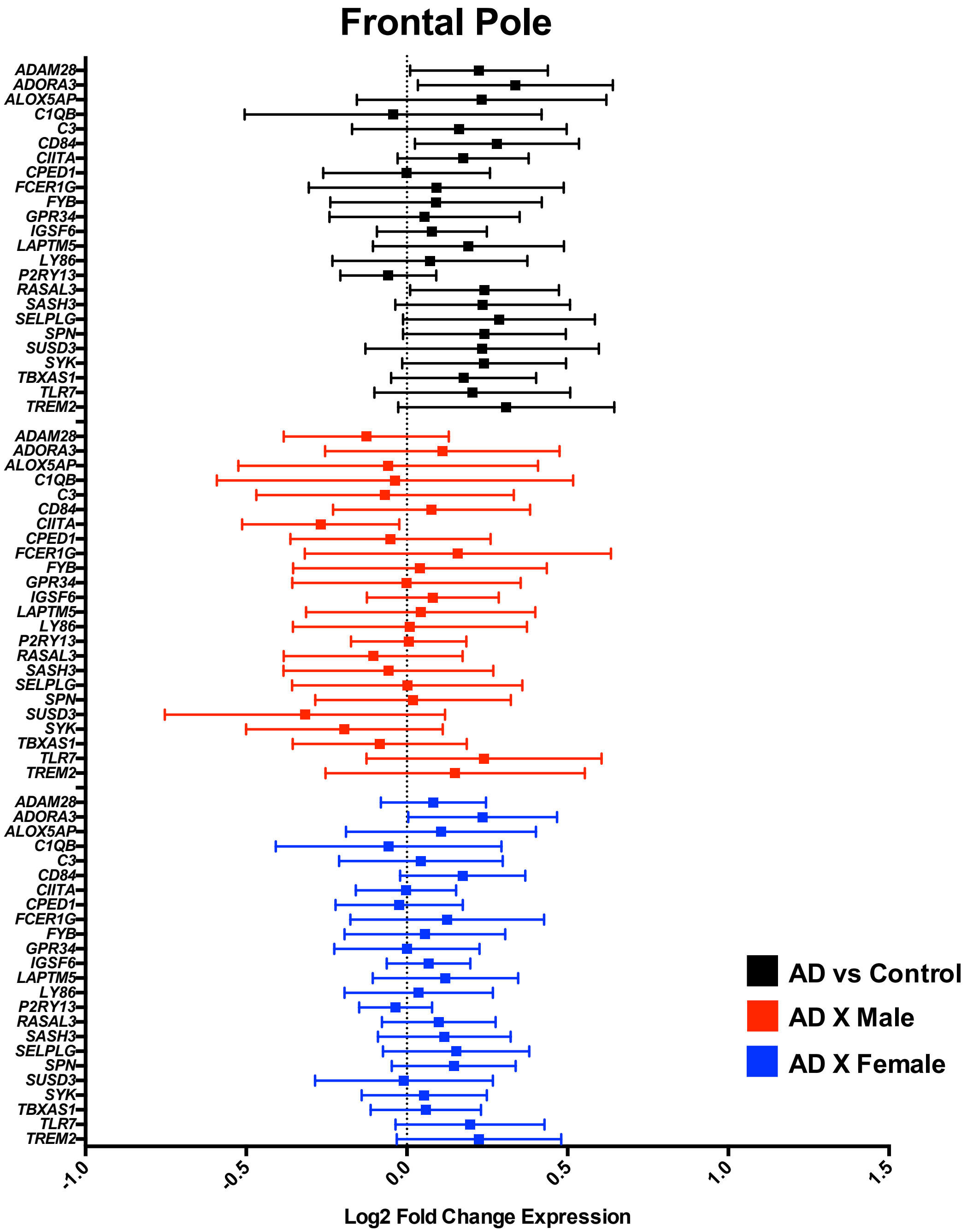

Frontal Pole
ADAM28
ADORA3
ALOX5AP
C1QB
C3
CD84
CIITA
CPED1
FCER1G
FYB
GPR34
IGSF6
LAPTM5
LY86
P2RY13
RASAL3
SASH3
SELPLG
SPN
SUSD3
SYK
TBXAS1
TLR7
TREM2
-1.0
-0.5
0.0
0.5
1.0
1.5
Log2 Fold Change Expression
AD vs Control
AD X Male
AD X Female

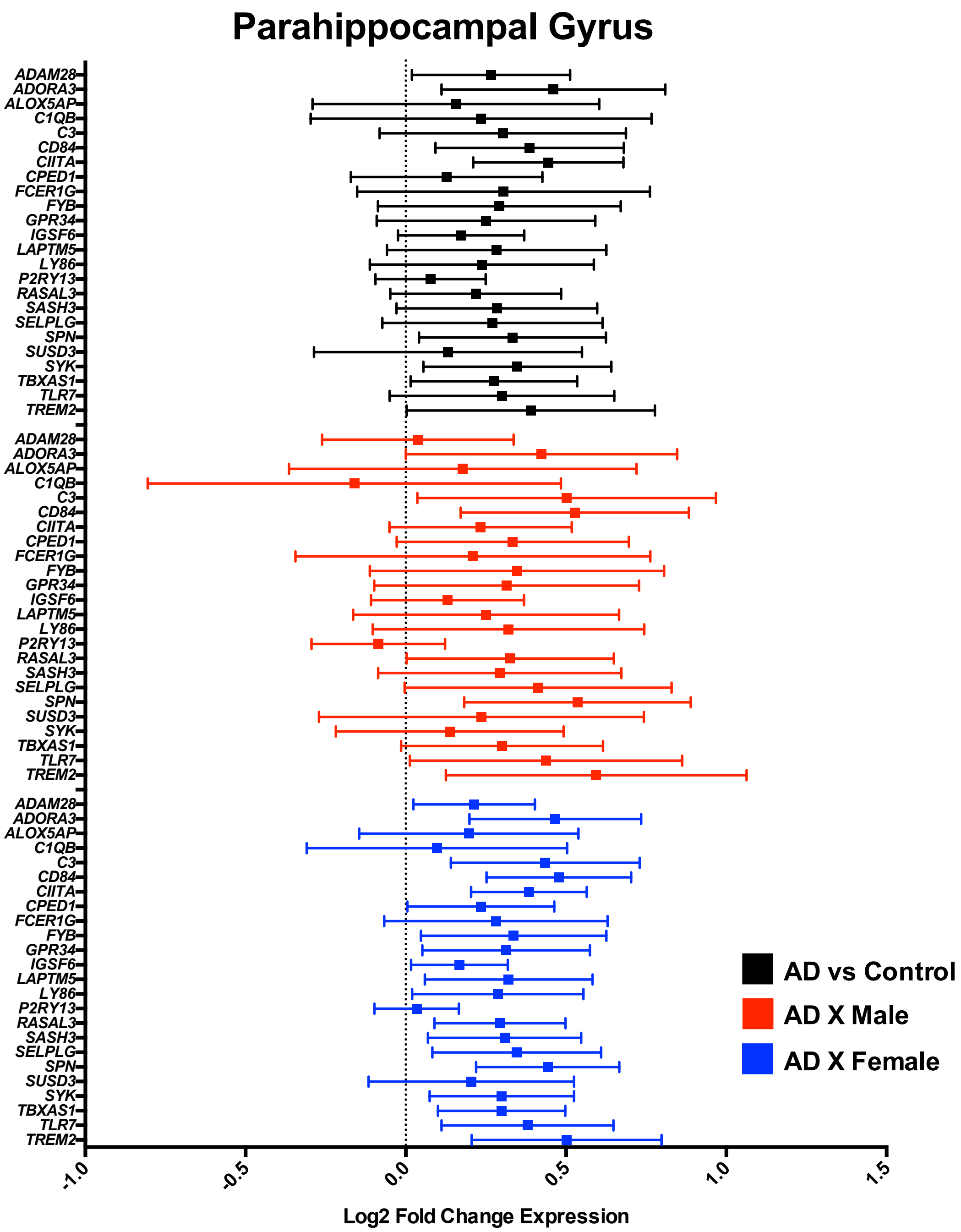

Parahippocampal Gyrus
ADAM28
ADORA3
ALOX5AP
C1QB
C3
CD84
CIITA
CPED1
FCER1G
FYB
GPR34
IGSF6
LAPTM5
LY86
P2RY13
RASAL3
SASH3
SELPLG
SPN
SUSD3
SYK
TBXAS1
TLR7
TREM2
AD vs Control
AD X Male
AD X Female
-1.0
-0.5
0.0
0.5
1.0
1.5
Log2 Fold Change Expression

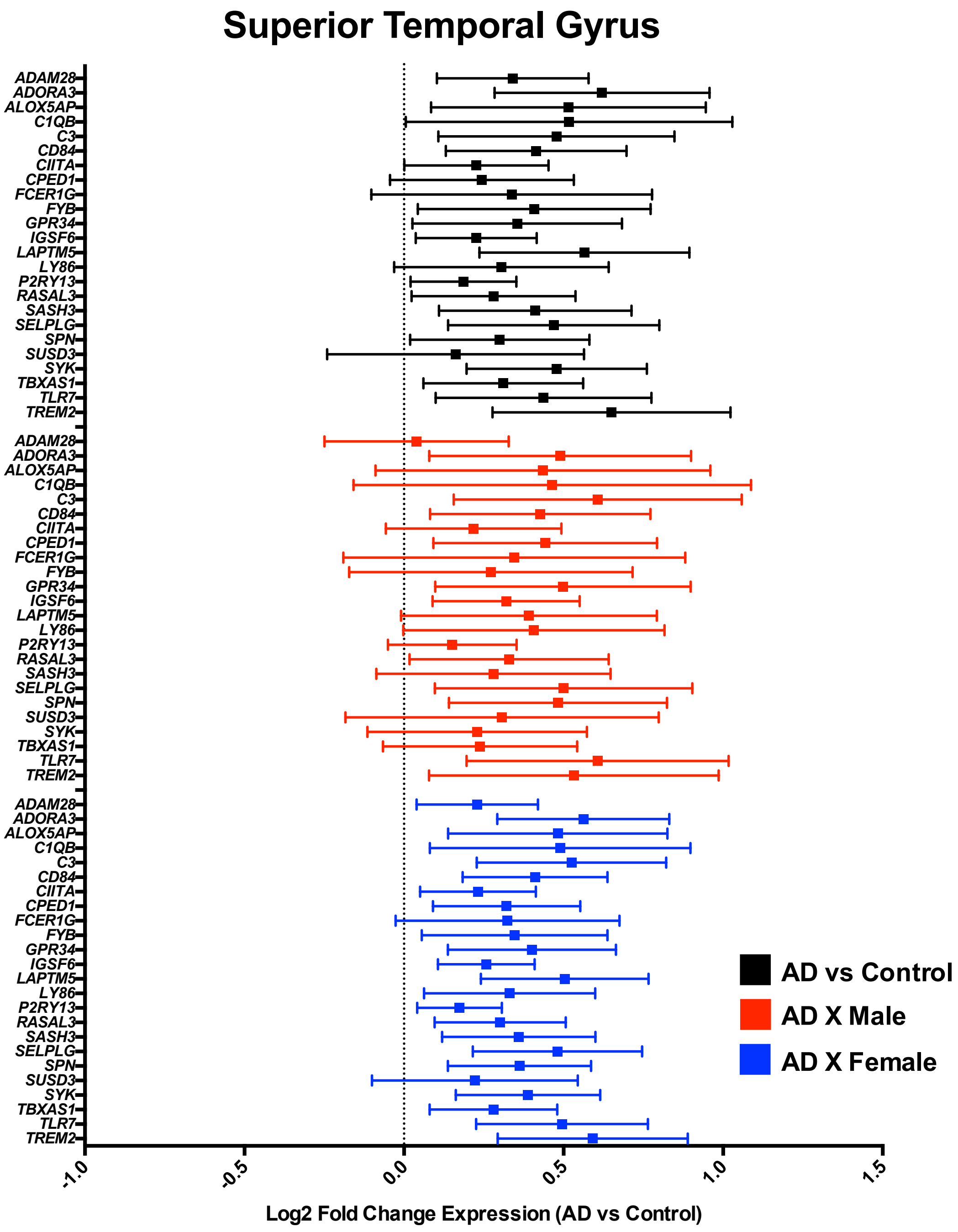

Superior Temporal Gyrus
ADAM28
ADORA3
ALOX5AP
C1QB
C3
CD84
CIITA
CPED1
FCER1G
FYB
GPR34
IGSF6
LAPTM5
LY86
P2RY13
RASAL3
SASH3
SELPLG
SPN
SUSD3
SYK
TBXAS1
TLR7
TREM2
AD vs Control
AD X Male
AD X Female
-1.0
-0.5
0.0
0.5
1.0
1.5
Log2 Fold Change Expression (AD vs Control)

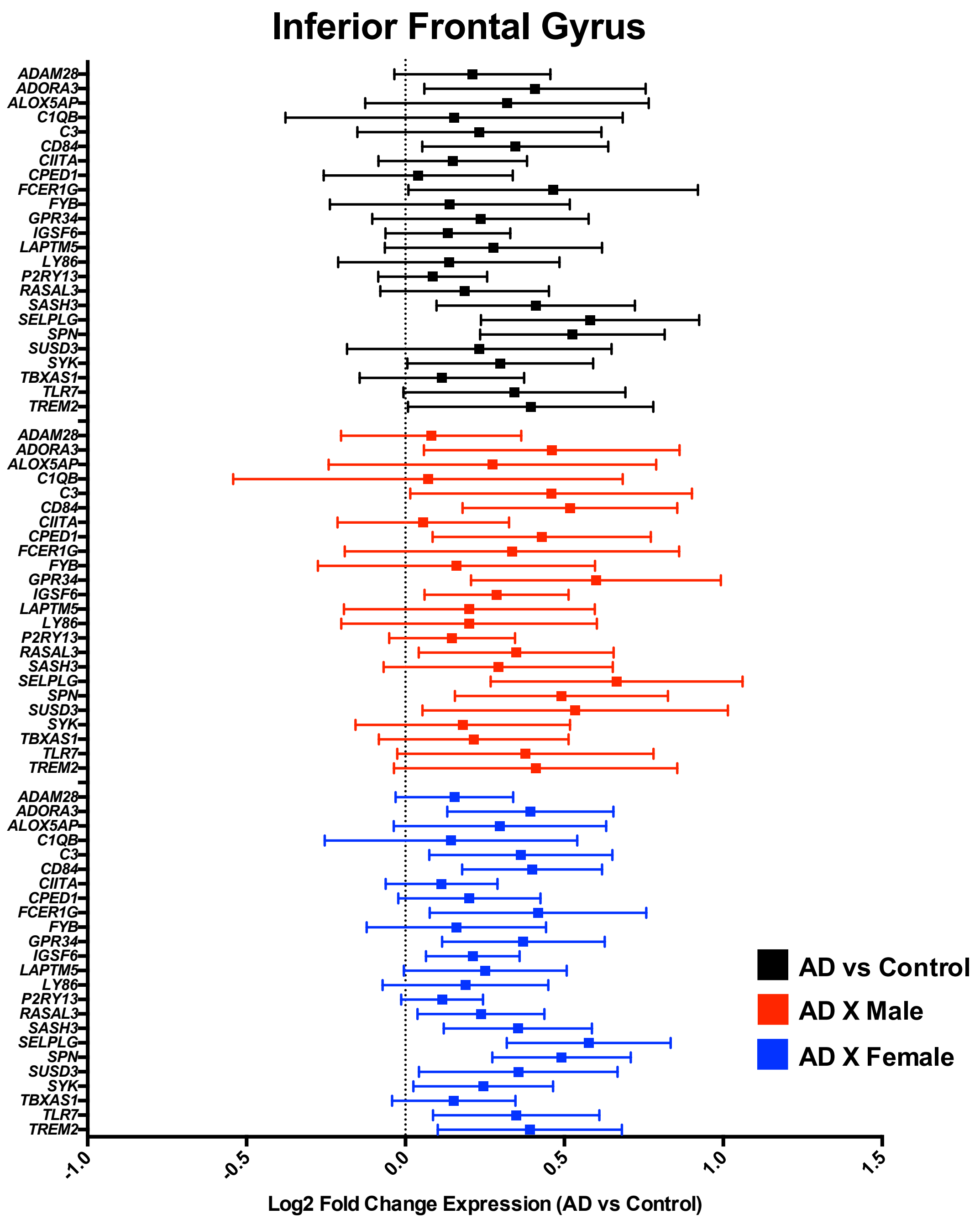

Figure S3. To explore the potential relationships between microglial genes and sex, we analyzed microglial gene expression testing for a statistical interaction between Alzheimer's disease diagnosis and sex (either male or female; e.g. Gene expression =

Diagnosis x Sex + covariates + (1|Donor)). The results shown in black at the top of each plot were previously presented in Figure 4 and Supplementary Figure 2 and are provided so the main effect of diagnosis can be compared to the interaction effect between sex and diagnosis. The results of these analyses are shown in red for males and blue for females. Overall, there was slightly higher number of significant results for the diagnosis x female interaction than the diagnosis x male interaction (see temporal cortex, parahippocampal gyrus, and superior temporal gyrus), but this effect appears subtle. For the presented analyses, we evaluated the entire gene set when possible, omitting specific microglial genes only when expression data was not available.
