# Supplementary material for: The Transcriptional Landscape of Microglial Genes in Aging and Neurodegenerative Disease
Source: Front Immunol. 2019 Jun 4;10:1170. doi: 10.3389/fimmu.2019.01170 (PMC6557985; doi:10.3389/fimmu.2019.01170)
Supplement: Supplementary file 5 [file Image_4.pdf]

Figure S4. FUMA GWAS analysis results

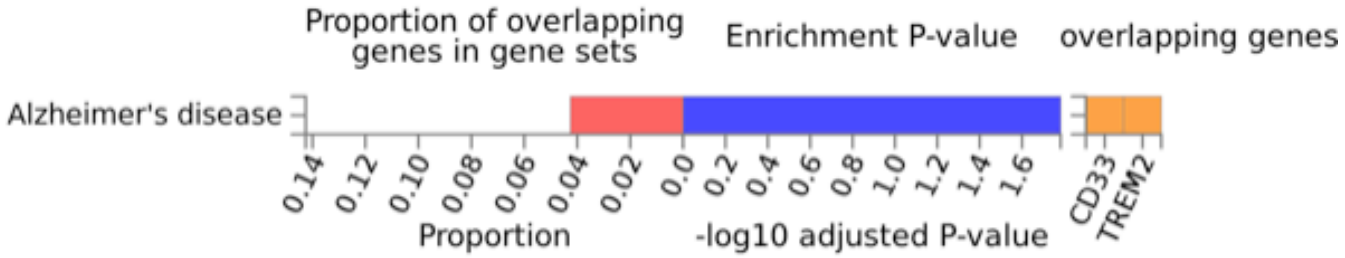

Figure S4. Results from FUMA GWAS for the 30 microglial genes of interest demonstrate significant enrichment for Alzheimer's disease risk genes (raw p-value = 8.87 E-6, FDR corrected p-value = 0.017).
